# Comparative Study on Physiological Responses and Gene Expression of Bud Endodormancy Release Between Two Herbaceous Peony Cultivars (*Paeonia lactiflora* Pall.) With Contrasting Chilling Requirements

**DOI:** 10.3389/fpls.2021.772285

**Published:** 2022-02-02

**Authors:** Xiaobin Wang, Runlong Zhang, Qiaoyu Huang, Xiaohua Shi, Danqing Li, Lingmei Shao, Tong Xu, David P. Horvath, Yiping Xia, Jiaping Zhang

**Affiliations:** ^1^Genomics and Genetic Engineering Laboratory of Ornamental Plants, Department of Horticulture, College of Agriculture and Biotechnology, Zhejiang University, Hangzhou, China; ^2^State Key Laboratory of Subtropical Silviculture, School of Forestry and Biotechnology, Zhejiang A&F University, Hangzhou, China; ^3^Zhejiang Institute of Landscape Plants and Flowers, Hangzhou, China; ^4^Agricultural Research Service, United States Department of Agriculture, Washington, DC, United States

**Keywords:** bud endodormancy release, reactive oxygen species, abscisic acid, *Paeonia lactiflora*, warm winter, geophyte, underground bud, gene expression

## Abstract

With the global temperature increase, diverse endogenous factors and environmental cues can lead to severe obstacles to bud endodormancy release for important economic plants, such as herbaceous peony (*Paeonia lactiflora* Pall.). Knowing the underlying mechanism in bud endodormancy release is vital for widely planting herbaceous peony at low latitudes with warm winter climates. A systematic study was carried out between the southern Chinese cultivar ‘Hang Baishao’ with low-chilling requirement (CR) trait and the northern cultivar ‘Zhuguang’ with high-CR trait. Peony buds were sampled at regular intervals under natural cold during the crucial bud endodormancy release stage. Physiology and morphology of the buds were observed, and the roles of reactive oxygen species (ROS) and relevant genes in the regulation of bud endodormancy release were also highlighted, which has been rather rare in previous bud dormancy studies of both herbaceous and tree peonies. The expression of the starch metabolism- and sucrose synthesis-related genes *PlAMY PlSPS* and *PlSUS* was lower in the high-CR ‘Zhuguang’ and corresponded to a lower content of soluble sugars. The expression of polyamine oxidase gene *PlPAO2* correlated with a higher level of hydrogen peroxide (H_2_O_2_) in high-CR ‘Zhuguang’ than in low CR ‘Hang Baishao’ during bud endodormancy. Expression of *PlMAPKKK5*, an intermediate gene in the abscisic acid (ABA) response to ROS signaling, correlated with ROS levels and ABA content. We present the hypothesis that accumulation of ROS increases ABA content and decreases GA_3_ content and signal transduction leading to reduced expression of *PlSVP* and *PlSOC1*. Reduced cell division and increased cellular damage which probably blocked bud endodormancy release were also observed in high-CR ‘Zhuguang’ through histological observation and related genes expression. This study provides a comparative analysis on physiological responses and gene expression patterns of bud dormancy of geophytes in an increasingly unsuitable environment.

## Introduction

Bud dormancy is an important physiological phenomenon that allows perennial plants to endure harsh environmental conditions during winter (Azeez et al., 2021), and generally includes three types: paradormancy, endodormancy, and ecodormancy (Falavigna et al., 2018). Paradormancy is generally caused by apical dominance, and the growth of lateral buds is suppressed by actively growing parts of the plant (Horvath et al., 2003). Prolonged exposure to short days or low temperatures in late autumn induce the establishment of endodormancy in buds. Endodormancy is growth cessation controlled by internal physiological factors, and in this stage, the buds cannot sprout and elongate without meeting chilling requirements (CRs), even under favorable conditions (Yordanov et al., 2014; Zhu et al., 2020). Sufficient CRs progressively lead to endodormancy release, following which growth arrest is primarily maintained by low temperatures in the winter. As growth arrest is controlled by external environmental factors, buds are described as being in ecodormancy. In ecodormant state, warm temperatures in the spring are necessary for bud burst (Yang et al., 2021). Global warming has resulted in countless perennial plants facing incomplete endodormancy associated with insufficient CR and poses obstacles to bud sprouting (Prudencio et al., 2020; Wang et al., 2021). This hinders the ability of these plants to grow well and consequently reduces their economic value after warm winters, especially in northern cultivars grown at lower latitudes (Zhang et al., 2017; Jewaria et al., 2021). Therefore, elucidating the mechanism controlling endodormancy release may help researchers address the problems caused by warming winter climates.

The induction and release of bud dormancy are controlled by various regulators (Horvath et al., 2008; Chao et al., 2017; Singh et al., 2017). Currently, it is widely accepted that bud dormancy is controlled by the balance of phytohormones, especially abscisic acid (ABA) and gibberellins (GAs), which show an antagonistic effect on dormancy process regulation (Singh et al., 2018; Yang et al., 2020). The establishment of endodormancy is accompanied by an increasing ABA content and a decrease in GAs, while endodormancy release is dependent on sufficient GA concentrations (Li Z. et al., 2020). Bud dormancy is affected not only by the contents of ABA and GAs but also, more importantly, by the ABA signal transduction pathway (Dong et al., 2019). In the ABA signal transduction pathway, ABA combined with ABA receptor pyrabactin resistance 1 protein (PYR1)/PYR1-like (PYL) could inhibit the enzyme activity of protein phosphatase 2C (PP2C). Inhibition of PP2C activity enables activation of SNF1-RELATED KINASE 2 (SnRK2) protein kinases, which then activates downstream transcription factors that induce ABA-responsive gene expression (Shu et al., 2016). In the GA signal transduction pathway, the DELLA protein inhibits bud growth and development of plants in the absence of GA (Yuxi et al., 2021). In the GA signal transduction pathway, the bud growth and development of plants are inhibited by the DELLA protein in the absence of GA (Yuxi et al., 2021). Binding of bioactive GA to the *GA-INSENSITIVE DWARF1* (*GID1*) receptor, which in turn promotes the interaction between *GID1* and DELLAs, subsequently leads to the degradation of DELLA proteins and consequently relieves their suppressive effect, thereby controlling seed dormancy (Ariizumi et al., 2013; Tuan et al., 2021). In buds, it has been demonstrated that ABA can close symplastic communication to the meristems (Tylewicz et al., 2018) and GA signaling is required for re-opening these channels (Rinne et al., 2011).

The ability to break endodormancy even under high winter temperatures while still ensuring normal bud dormancy progress is a crucial trait for perennial plants to adapt to a global warming trend (Baumgarten et al., 2021; Yang et al., 2021). One particular pathway seems central and is almost always highlighted in breaking bud dormancy: the response to oxidative stress, especially reactive oxygen species (ROS) (Liao et al., 2021). ROS function as a signaling molecule in plants, acting as a regulator of plant growth and development, dormancy regulation, hormone signaling, and responses to biotic and abiotic stresses (Balazadeh et al., 2012; Raja et al., 2017). Evidence is emerging that ROS, especially (hydrogen peroxide) H_2_O_2_, together with plant hormones are part of the signaling network involved in dormancy release (Barba-Espin et al., 2011; Oracz and Karpinski, 2016). This fits with suggestions that inhibited catalase (CAT) activity (Liang et al., 2019) and increased level of H_2_O_2_ stimulate bud dormancy in grape buds (Meitha et al., 2015; Sudawan et al., 2016). Although several lines of evidence indicate that ROS (mainly is H_2_O_2_) promote dormancy release, with exception of some work on grape bud dormancy (Pérez et al., 2008; Zheng et al., 2015), there is little information establishing a direct link between ROS and the release of bud dormancy by affecting the ABA content under stressful environments.

MIKC*^C^*-type MADS-box genes encode transcription factors that have key roles in controlling bud dormancy and flowering time in plants (Kumar et al., 2016, 2021). Several crucial MIKC*^C^* genes, specifically *SHORT VEGETATIVE PHASE LIKE* (*SVP*)*/Dormancy Associated MADS-Box* (*DAM*), *FLOWERING LOCUS C* (*FLC*) and *SUPPRESSOR OF OVEREXPRESSION OF CO 1* (*SOC1*), have been found to be vital for releasing bud dormancy (Falavigna et al., 2018; Singh et al., 2019; Yang et al., 2021). In poplar, *SVL* (*SVP* like), which is induced by ABA and forming a positive feedback loop with ABA signaling, is a crucial transcription factor suppressing the release of bud endodormancy (Singh et al., 2019). Meanwhile, *SVL* also inhibits the expression of *GA 20-oxidase 1* (*GA20ox1*) and *GA20ox2* and increases the expression of *GA2ox8*, thus decreasing the level of GAs and deepening bud endodormancy (Wu et al., 2017). *SOC1* is a vital flowering signal integrator that promotes bud dormancy and flowering (Li et al., 2019). *SVP* directly binds to the CArG box of the *SOC1* promoter, represses the expression of *SOC1* and thus delays flowering in Arabidopsis (Li et al., 2008). Therefore, *SVP*/*SVL* and *SOC1* can interact and integrate the metabolism of ABA and GA to maintain or release endodormancy (Yang et al., 2021).

Herbaceous peony (*Paeonia lactiflora* Pall.) is a world-famous ornamental geophyte with underground buds (Bian et al., 2020). Annual life cycle of herbaceous peony shows a typical characteristic in underground bud dormancy, which could be used as crucial material for studying bud dormancy of geophytes. Buds of herbaceous peony begin to differentiate into flora organs in October, then develop throughout the winter and sprout in early spring. Afterward, the buds elongate rapidly and enter the state of vegetative growth. When the aboveground parts wither in later September or early October, the underground buds enter endodormancy and release it after receiving sufficient CRs around January (Halevy et al., 2005). Enriching the research on the dormancy of underground buds could help fully elucidate the bud dormancy mechanism of geophytes, and then promote the cultivation of herbaceous peony with a short endodormancy duration (low-CR trait) at low latitudes with warm winters (Prudencio et al., 2020).

Here, a systematic study was carried out comparing the introduced northern Chinese cultivar ‘Zhuguang’ with high-CR trait, and the native southern Chinese cultivar ‘Hang Baishao’ with low-CR trait at low latitudes under warm winters. The aim of this study was to find the key factors that may induce obstacles to bud endodormancy release caused by warm winters and lay a foundation for further study of molecular mechanisms of bud dormancy in herbaceous peony. In this study, systematic physiological and morphological observations were carried out during the crucial bud dormancy stages; the expression of the genes involved in key pathways was also analyzed. Finally, we developed a mechanistic model for investigating obstacles to bud endodormancy release of herbaceous peony caused by warm winters. Although this study is primarily observational and designed to obtain information needed to develop testable hypotheses, some interesting findings discovered in this study and several testable hypotheses throughout the discussion provide additional insights about the interaction between the environment and genotypes and suggestions for further research.

## Materials and Methods

### Plant Materials

**FIGURE 1:**
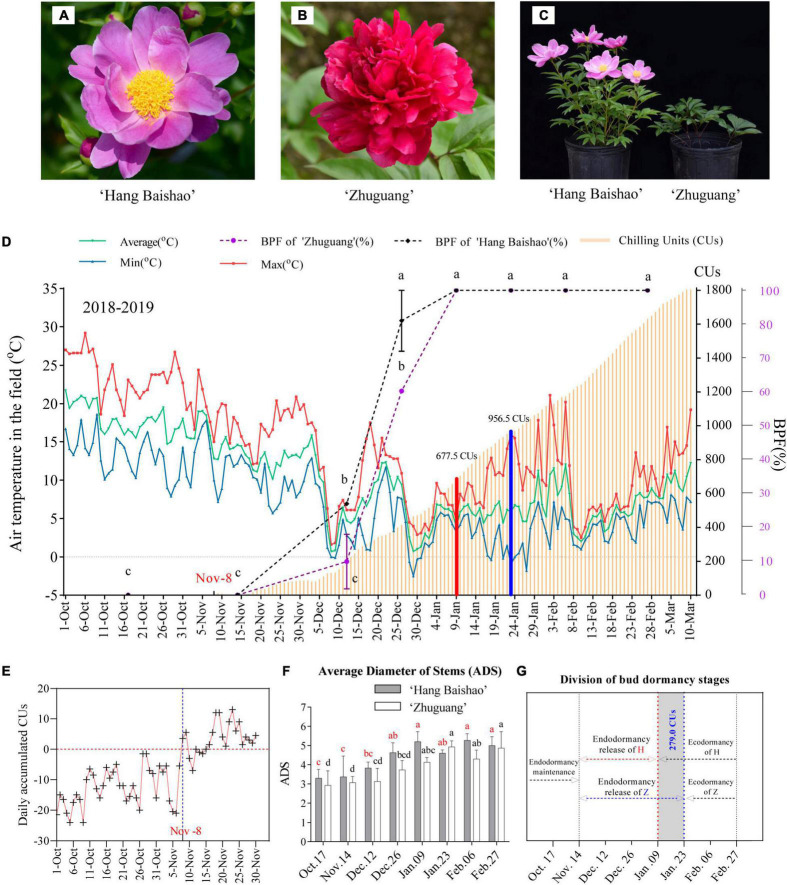
Plant materials, CR evaluation and morphological observations in this study. **(A,B)** Plant materials ‘Hang Baishao’ and ‘Zhuguang’. **(C)** Performance of the two cultivars on the transport date January 09 after regrowth in the glasshouse. **(D)** Air temperatures, BPF and CR evaluation in 2018–2019. The date in red (November 08) was the start date of the CR evaluation. The line chart represents BPF. The CR value was 677.5 CUs on January 09 (red bar) and 956.5 CUs on January 23 (blue bar) for ‘Hang Baishao’ and ‘Zhuguang,’ respectively. Error bars represent the standard deviation from three biological replicates, and different letters indicate significant differences (*P* < 0.05). Differences were compared among different move dates for each cultivar. **(E)** Daily accumulated CUs for determining the start time of CR evaluation. November 08 was the date on which the daily accumulated CUs first became positive. **(F)** Morphological index ADS of the two cultivars. **(G)** Division of bud dormancy stages of the two cultivars. Dashed lines indicate the division of bud dormancy stages, H represents ‘Hang Baishao’ and Z represents ‘Zhuguang.’ The gray shaded part represents the CR difference between the two cultivars, which was 279.0 CUs.

*Paeonia lactiflora* Pall. ‘Hang Baishao’ (native southern low-CR cultivar) (Figure 1A) and ‘Zhuguang’ (introduced northern high-CR cultivar) (Figure 1B) were selected herbaceous peony materials with contrasting responses to endodormancy release (Zhang et al., 2019a; Wang et al., 2020) (Figure 1C and Table 1). In the autumn of 2018, three-gallon pots of 4-year-old crowns of ‘Hang Baishao’ and ‘Zhuguang’ were placed in the Perennial Flower Resources Garden of Zhejiang University in Hangzhou (E 118°21′-120°30′, N 29°11′-30°33′), Zhejiang Province, China, where they grew under natural sunlight and standard maintenance before subsequent treatments.

**TABLE 1:** Details of *Paeonia lactiflora* ‘Hang Baishao’ and ‘Zhuguang.’

Names	Origins	Latitudes	Flower types	CRs (CUs)	CR traits
‘Hang Baishao’	Zhejiang, China	N 28°–29°	Single	685.5–746.0	Low-CR
‘Zhuguang’	Shandong, China	N 34°–35°	Crown	1017.0–1062.0	High-CR

*The latitudes here refer to the latitude at which these two cultivars are concentrated for cultivation in China. CRs of the two cultivars were obtained in the previous study (Wang et al., 2020).*

### Experimental Design and Sampling

Sufficient potted crowns of ‘Hang Baishao’ and ‘Zhuguang’ were subjected to natural chilling in the field during the winters of 2018–2019. Previous records about winter conditions and chill accumulation in 2012–2014, 2016–2018, please refer to Wang et al. (2020). On October 17, November 14, December 12, December 26, January 09, January 23, February 06, and February 27, which covered the bud endodormancy, ecodormancy and bud break stages of herbaceous peony in Hangzhou from 2018 to 2019, plants (three biological replicates and three potted crowns per replicate) were moved to a glasshouse for morphological observations (25–15°C day/night, 80% relative humidity, regular watering and fertilizer applications). At the same time, underground buds were sampled from another group of potted crowns in the filed under nature cold on each move date. Sampled buds for physiological measurements and gene expression analysis (three biological replicates and nine plants per replicate) were washed with distilled water, frozen immediately in liquid nitrogen and stored at −80°C until use, while buds for paraffin section were fixed with FAA (formalin, acetic acid and alcohol).

### Morphological Observations

Morphological indices were evaluated for potted crowns (three biological replicates, three potted crowns per replicate) of ‘Hang Baishao’ and ‘Zhuguang’ after transported into the glasshouse (Table 2). The date on which most indices did not increase or decrease significantly, even if more chilling duration exposure occurred, was defined as endodormancy release (Wang et al., 2020).

**TABLE 2:** Full names and definitions of the seven morphological indices for potted crowns of low-CR ‘Hang Baishao’ and high-CR ‘Zhuguang’ transported into the glasshouse.

Morphological indices	Full names	Definitions
WFS (w)	The number of weeks until the first plant sprouted.	Sprouting was defined as bud scales opened and tender shoots/leaves emerged.
WAS (w)	The number of weeks until all plants sprouted.	If a plant in a pot grew at least one sprouted bud, we defined it as a sprouted plant.
BPF(%)	The bud break percentage 5 weeks after transported to the glasshouse.	
ANS	The average number of mature and normal stems.	A stem with at least three compound leaves and plant height longer than 20 cm as a mature and normal stem.
ADS (mm)	The average diameter of mature and normal stems.	Diameter of mature and normal stem at five cm from the ground of the plant.
APH (cm)	The average plant height.	The distance (cm) from the ground to the top of the plant.
APW (cm)	The average plant height and width.	Maximum width (cm) of aboveground projection of the plant.

*The indices ANS, ADS, APH, and APW were measured after the plants moved to glasshouse and regrowth to a stable state.*

### Bud Morphology Analyses

A subsample of buds from each time point were separately fixed in FAA and embedded in paraffin. Sections were cut at 8–10 μm thickness. Safranine fast green staining was used to observe cell division and vascular bundles. Sections were put into xylene for 5 min to mounted the tissue section with neutral balsam. After sealing with neutral balsam, the sections were observed under a microscope (NIKON ECLIPSE E100, Tokyo, Japan).

### Chilling Requirement Evaluation

The Utah (UT) model was determined to be the optimal model for CR evaluation of herbaceous peony at low latitudes according to our previous study and thus was selected to evaluate the CRs of ‘Hang Baishao’ and ‘Zhuguang’ (Richardson et al., 1974; Cesaraccio et al., 2004; Wang et al., 2020). The start time of the UT model was defined as 0:00 am on the day when the daily accumulated chill units (CUs) increased and first became positive after October. The cumulative CUs were calculated from the start time to the last observation day (March 10) in the field. CRs of the two cultivars were the specific accumulated CUs corresponded to the dates that released endodormancy, respectively. Air temperatures were automatically recorded hourly with a GM200-TH temperature and humidity recorder (Zhituo Instruments Limited Company, Hangzhou, China) in the field.

### Carbohydrate Concentration Measurements

Anthrone colorimetry was used for the determination of soluble sugar and starch contents. The relevant detailed procedures are introduced in detail in Supplementary Methods 1.

### Endogenous Hormone Quantitation

Endogenous hormone levels of buds were determined using an enzyme-linked immunosorbent assay (ELISA), and detailed procedures are introduced in Supplementary Methods 2.

### Reactive Oxygen Species Related Physiological and Biochemical Measurements

An underground bud sample (0.3 g) was fully ground with 3 mL phosphate-buffered saline (1X, PH = 7.4). Then, the homogenate was transferred to a 10 mL centrifuge tube and centrifuged at 4°C and 3,500 rpm centrifugation for 10 min. Afterward, the supernatant was collected and the indices were measured using detection kits purchased from Nanjing Jiancheng Bioengineering (Nanjing, China) Co., Ltd. and following the manufacturer’s instructions (Zhao et al., 2020). Specifically, The contents of malondialdehyde (MDA) and soluble protein were measured using thiobarbituric acid and Coomassie brilliant blue methods, respectively, according to the MDA assay kit (A003-3) and soluble protein assay kit (A045-2). The H_2_O_2_ concentrations were measured using a H_2_O_2_ Detection Kit (A064-1). For enzyme assays, Superoxide dismutase (SOD) activity was measured using a SOD Detection Kit (A001-3). Catalase (CAT) activity was measured using a CAT Detection Kit that detects degradation of H_2_O_2_ at 405 nm (A007-1). Peroxidase (POD) activity was detected with a POD Detection Kit for plants (A084-3). Three biological replicates of each assay were performed.

### Screening of the Key Genes Involved in the Regulation of Bud Dormancy

In combination with previous studies on bud dormancy and physiological measurements in this study, we focused on metabolic pathways that play important roles in the regulation of bud dormancy, such as carbohydrate metabolism, hormone signal transduction, ROS signaling, and MADS-box gene-related pathways. In 2017–2018, we have performed transcriptome sequencing on buds of ‘Hang Baishao’ and ‘Zhuguang’ under natural cold. The experimental period was from fall to winter, with the specific dates for sampling being October 17, 2017, November 14, 2017, December 12, 2017, December 26, 2017, January 09, 2018, January 23, 2018, February 06, 2018, and February 27, 2018. Chill accumulation of each sampling date from October 17, 2017–February 27, 2018 evaluated by UT model were: – (no result), −38.5, 277.0, 479.5, 746.0, 980.0, 1062.0, and 1434.5 CUs, respectively (Wang et al., 2020). Total RNA was extracted from the sampled buds (three biological replicates, three plants per replicate) and the quality and quantity of the RNA were checked. Afterward, cDNA libraries of the two cultivars were constructed and sequenced using Illumina Hiseq X-Ten platform, respectively. Transcripts were obtained by *de novo* assembly according to the method of Liu B. et al. (2020). According to the differentially expressed transcript (DET) annotation and FPKM expression values obtained based on the database of transcriptome sequences of ‘Hang Baishao’ and ‘Zhuguang’ (unpublished data), the key genes related to the above four metabolic processes were selected to carry out subsequent gene expression studies (Supplementary Table 1).

### Identification and Classification of Crucial MIKC*^C^*-Type MADS-Box Genes in *P. lactiflora*

To identify crucial MIKC*^C^*-type MADS-box genes *SVP*, *SOC1*, *AP1*, and *FLC* in *P. lactiflora*, a profile hidden Markov model (HMMER) of the SRF-TF domain (Pfam accession: PF00319) was obtained from the Pfam database^1^. BLAST results obtained were further filtered with a query coverage of 90%, and the best hits were retrieved. Selected *P. lactiflora* proteins are provided in Supplementary Table 2. In addition, the following species were also selected to investigate their evolutionary relationships: *Amborella trichopoda* (scaffold), *Arabidopsis thaliana* (At), *Vitis vinifera* (VIT), *Oryza sativa* (LOC_Os), *Prunus persica* (Prupe.), *Populus trichocarpa* (Potri.), *Euphorbia esula* (PJAD), *Actinidia chinensis* (Actinidia) and *Paeonia suffruticosa* (psu). The sequences were aligned using MAFFT (Katoh and Standley, 2013) with default parameters to assign the four putative MIKC*^C^*-type MADS-box genes to specific gene subfamilies. A maximum-likelihood phylogenetic tree was constructed using FastTree software using the JTT + CAT model (Price et al., 2009).

### Total RNA Extraction and Quantitative Real-Time PCR Analysis

Total RNA was isolated from underground buds via an RNAprep Pure Plant Kit (polysaccharide- and polyphenolic-rich) (Tiangen, Beijing, China). The quality and quantity of RNA and the validity of the reference gene *Alpha-tubulin* (*ATUBA*) used for qRT–PCR were introduced in Supplementary File 1. Reverse transcription was performed with a PrimeScript RT Reagent Kit (Takara Biotechnology, Dalian, China). Primers and candidate genes were listed in Supplementary Table 1. qRT–PCR was conducted with SYBR Premix Ex Taq (Takara Biotechnology). The PCR procedure used was according to the manufacturer’s instructions. Three biological replicates were prepared per sample, and the 2^–ΔΔCT^ method was used to calculate the gene expression level (Livak and Schmittgen, 2001).

### Statistical Analysis

All experiments in this study were conducted in accordance with a completely randomized design. One-way analysis of variance (ANOVA) was used to compare differences among different indices or treatments via SPSS 26.0 (IBM Corp., Armonk, NY, United States), with a probability value of *P* < 0.05 considered significant. GraphPad Prism 9.0 (GraphPad Software, Inc., La Jolla, CA, United States) and Tbtools Software (Chen et al., 2020) were used for figure construction.

## Results

### Chilling Requirement Evaluation of the Two Cultivars

With the decrease in air temperatures during autumn to winter (Figure 1D), the daily accumulated CUs calculated according to the UT model increased correspondingly (Figure 1E). November 08 was the day when the daily accumulated CUs first became positive after October (Figure 1E) and represented the start time for the calculation of chill accumulation. Cumulative CUs increased gradually after November 08, and decreased slightly around December 03 due to raised temperatures, then continued to increase until the last observation day in the field (Figure 1D). The end time of CR evaluation was based on morphological indices. The BPF, APH, APW, ANS, and ADS of the two cultivars increased while the WFS and WAS decreased gradually and then stabilized over time. Most of the indices for low CR ‘Hang Baishao’ were stable on January 09, while those for high-CR ‘Zhuguang’ were stable on January 23 (Figure 1F and Table 3). Thus, January 09 and January 23 were determined as the endodormancy release (CR fulfillment) dates for low CR ‘Hang Baishao’ and high-CR ‘Zhuguang,’ respectively (Wang et al., 2020). Low CR ‘Hang Baishao’ fully broke endodormancy when CR reached 677.5 CUs, 2019, while high-CR ‘Zhuguang’ required 956.5 CUs for full endodormancy breaking (Figure 1D).

**TABLE 3:** Morphological observations of the two cultivars after moved into glasshouse under natural chilling treatments in 2018–2019.

Name	Move dates	APH (cm)	APW (cm)	ANS	WFS (w)	WAS (w)
H	October 17	24.40 ± 2.38d	27.73 ± 5.28e	2.33 ± 0.58c	20.00	–*^X^*
	November 14	25.73 ± 0.86d	29.37 ± 4.38e	1.27 ± 0.25c	16.00	–
	December 12	38.07 ± 4.97c	37.00 ± 5.10d	2.00 ± 0.70c	3.00	–
	December 26	44.43 ± 9.55bc	47.07 ± 2.75c	4.10 ± 0.35b	3.00 ± 0.00a	6.00 ± 0.00 a
	January 09	52.50 ± 2.09ab	54.00 ± 1.65b	5.90 ± 1.93a	2.00 ± 0.00b	4.00 ± 0.00 b
	January 23	44.83 ± 2.34bc	38.13 ± 2.83d	6.43 ± 0.51a	1.00 ± 0.00c	2.00 ± 0.00 c
	February 06	57.20 ± 3.87a	61.17 ± 2.11a	5.33 ± 0.35ab	1.00 ± 0.01c	2.00 ± 0.00 c
	February 27	58.37 ± 4.45a	54.23 ± 1.15b	5.57 ± 1.25ab	1.00 ± 0.02c	1.00 ± 0.00 d

**Name**	**Move dates**	**APH**	**APW**	**ANS**	**WFS**	**WAS**

Z	October 17	15.40 ± 4.65e	23.93 ± 7.28c	1.50 ± 0.50d	28.00	–
	November 14	16.27 ± 1.65e	19.43 ± 2.72c	1.17 ± 0.29d	23.00	–
	December 12	26.27 ± 3.29d	33.10 ± 1.83b	1.77 ± 0.50cd	5.00	–
	December 26	28.33 ± 4.41cd	37.67 ± 4.63b	2.90 ± 0.85bc	3.00 ± 0.01a	8.33 ± 4.62 a
	January 09	41.83 ± 0.61a	56.47 ± 5.62a	4.43 ± 1.21a	2.00 ± 0.00b	3.00 ± 0.00 b
	January 23	37.30 ± 0.46ab	52.67 ± 1.92a	3.53 ± 0.40ab	2.00 ± 0.00b	2.00 ± 0.00 b
	February 06	38.53 ± 0.35ab	53.53 ± 4.29a	4.20 ± 0.17a	1.00 ± 0.00c	2.00 ± 0.00 b
	February 27	34.27 ± 6.83bc	49.70 ± 4.74a	3.43 ± 0.81ab	1.00 ± 0.01c	1.00 ± 0.00 b

*^X^The morphological data were non-existent because only partial plants sprouted finally due to insufficient chill accumulation. APH and APW, the average plant height and width, respectively; ANS, the average number of mature and normal stems; WFS, the number of weeks until the first plant sprouted in the glasshouse; WAS, the number of weeks until all plants sprouted in the glasshouse. H represents ‘Hang Baishao’ and Z represents ‘Zhuguang.’ All values are means ± standard deviation, different letters indicate significant differences (P < 0.05). Differences were compared among different move dates for each cultivar.*

**FIGURE 2:**
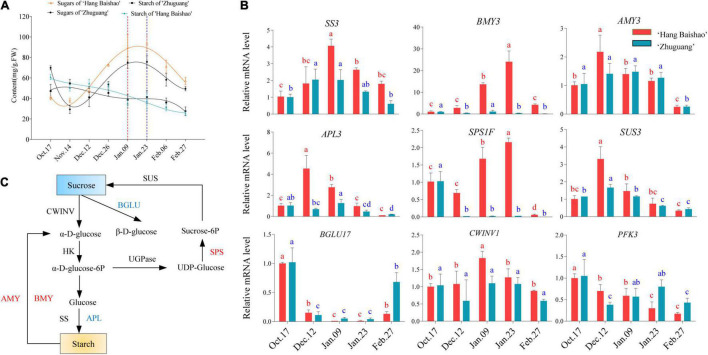
Starch and sucrose metabolism and the expression of related key genes during bud dormancy. **(A)** Concentrations of soluble sugar and starch in the two cultivars. The data were fitted with a third-order polynomial by GraphPad Prism 9.0. The dashed red line indicates the bud endodormancy release date of low CR ‘Hang Baishao,’ and the dashed blue line indicates the bud endodormancy release date of high-CR ‘Zhuguang.’ **(B)** Relative mRNA levels related to sucrose and starch metabolism analyzed by qRT–PCR. **(C)** Summary of different factors in the starch and sucrose metabolism pathways during bud dormancy. Red indicates upregulation, and blue indicates downregulation. Error bars represent standard deviation from three biological replicates and different letters indicate significant differences (*P* < 0.05). Differences were compared among different move dates for each cultivar.

Bud morphological indices and trends in carbohydrate content are closely related to the dormancy process and are often used to divide bud dormancy stages. In this study, morphological indices, such as the WFS, APW and APH, changed little from October 17 to November 14 but changed significantly from November 14 to the CR fulfillment dates in both low CR ‘Hang Baishao’ and high-CR ‘Zhuguang’ (Table 3). This correlated with the trend for the sucrose and starch contents (Figure 2A). Thus, October 17, 2018, to November 14, 2018, was divided into the bud endodormancy maintenance stage in both cultivars (Figure 1G). November 14, 2018, to January 09, 2019, and January 23, 2019, were divided into the bud endodormancy release stages for low CR ‘Hang Baishao’ and high-CR ‘Zhuguang,’ respectively. Ecodormancy stages were established afterward in the two cultivars (Figure 1G).

### Natural and Histological Observations of Underground Buds

**FIGURE 3:**
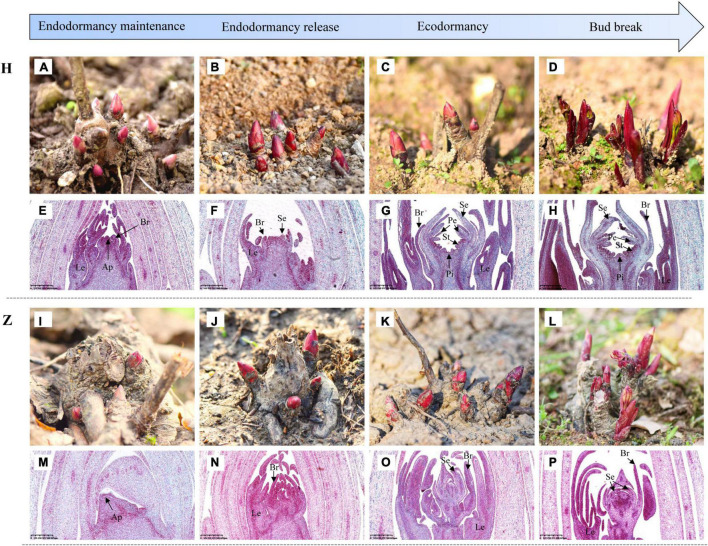
Observations of developmental growth stages and bud differentiation in *P. lactiflora*. **(A–D)** Developmental growth stages from bud endodormancy maintenance to bud break of buds in low CR ‘Hang Baishao.’ **(E–H)** Bud differentiation of buds in low CR ‘Hang Baishao.’ Le, Leaf primordia; Br, Bract primordia; Se, Sepal primordia; Pe, Petal primordia; St, Stamen primordia; Pi, Pistil primordia; and Ap, Apical meristem (Scale bar: 625 μm). **(I–L)** Developmental growth stages from bud endodormancy maintenance to bud break of buds in high-CR ‘Zhuguang.’ **(M–P)** Bud differentiation of buds in high-CR ‘Zhuguang’ (scale bar: 625 μm). H represents ‘Hang Baishao’; Z represents ‘Zhuguang.’

Developmental growth stages from bud endodormancy maintenance to bud break of buds were observed continuously in low-CR ‘Hang Baishao’ (Figures 3A–D) and high-CR ‘Zhuguang’ (Figures 3I–L). Leaf primordia (Le) and bract primordia (Br) were observed during the endodormancy maintenance stage of low CR ‘Hang Baishao’ on October 17, 2018 (Figure 3E). The apical meristem differentiated into sepal primordia (Se) during the endodormancy release stage on December 12, 2018 and further differentiated into entire floral organs, including petal primordia (Pe), stamen primordia (St), and pistil primordia (Pi) after endodormancy release in low-CR ‘Hang Baishao’ (Figures 3F–H). However, bud differentiation was obviously blocked in high-CR ‘Zhuguang.’ Paraffin sections showed that only Le, Br, and Se were differentiated during ecodormancy stage on February 27, 2019 (Figures 3M–P).

### Carbohydrate Contents and Related Gene Expression

With the accumulation of natural chilling, the soluble sugar contents of the two cultivars declined during the endodormancy maintenance stage and then increased rapidly during the endodormancy release stage and peaked on January 23. Afterward, there was a sharp decline during ecodormancy (Figure 2A). However, the content of soluble sugar in the low-CR ‘Hang Baishao’ was higher than that in the high-CR ‘Zhuguang’ during the endodormancy release and ecodormancy stages (Figure 2A and Supplementary Table 3). In contrast, starch content continued to decrease during all dormancy stages in the two cultivars (Figure 2A).

The expression of the key genes involved in starch metabolic and sucrose synthesis pathways was also investigated (Figure 2C). The expression levels of *PlSS* and *PlAPL* were downregulated, while *PlAMY* was upregulated in both low CR ‘Hang Baishao’ and high-CR ‘Zhuguang’ during endodormancy (Figure 2B). *PlSPS* and *PlBMY* were upregulated in low-CR ‘Hang Baishao’, and the expression levels were obviously higher than those in high-CR ‘Zhuguang’ during endodormancy (Figure 2B and Supplementary Table 3). Nevertheless, the expression of *PlSUS*, *PlBGLU17* and *PlPFK3* was downregulated in the two cultivars during endodormancy (Figure 2B).

### Contents and Related Gene Expression of Abscisic Acid and GA_3_

**FIGURE 4:**
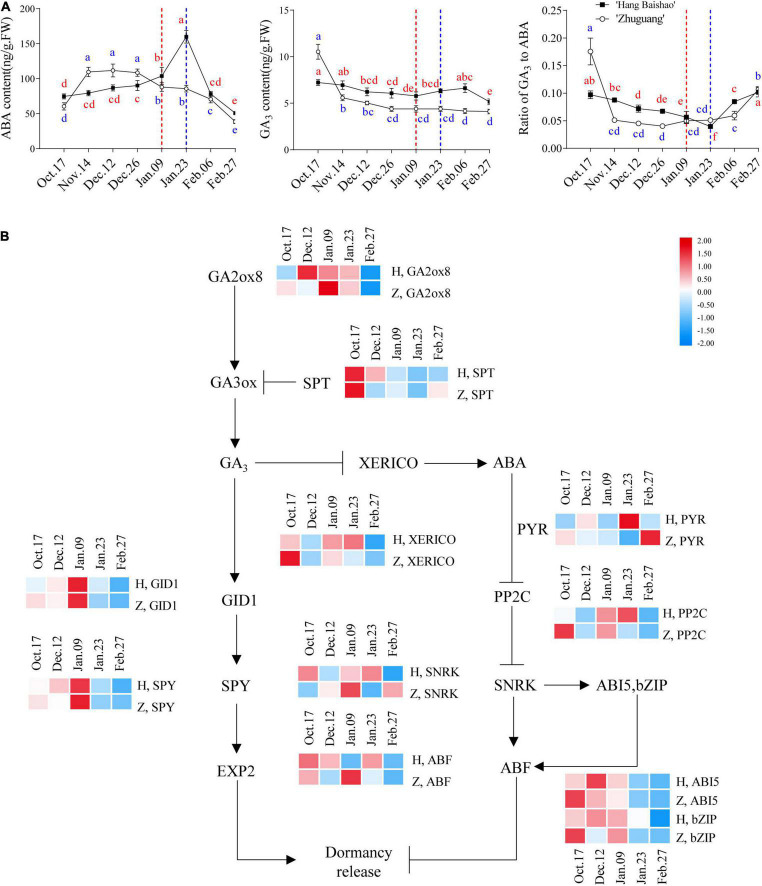
Expression patterns of genes involved in the GA_3_ and ABA signal transduction pathway and changes in concentrations of GA_3_ and ABA in ‘Hang Baishao’ and ‘Zhuguang’ during bud dormancy. **(A)** Concentrations of ABA and GA_3_ and ratio of GA_3_ to ABA in ‘Hang Baishao’ and ‘Zhuguang’ during bud dormancy. **(B)** Expression patterns of genes involved in the GA_3_ and ABA signal transduction pathway. The dashed red line indicates the bud endodormancy release date of low CR ‘Hang Baishao,’ and the dashed blue line indicates the bud endodormancy release date of high-CR ‘Zhuguang.’ H represents ‘Hang Baishao’; Z represents ‘Zhuguang.’ Error bars represent standard deviation from three biological replicates and different letters indicate significant differences (*P* < 0.05). Differences were compared among different move dates for each cultivar.

The ABA concentration of the high-CR ‘Zhuguang’ was significantly increased during endodormancy maintenance stage and maximized at the end of endodormancy maintenance stage, followed by a gradual reduction through to the end of the study. In low CR ‘Hang Baishao,’ the ABA content also increased gradually during early endodormancy release stage reaching levels similar to the peak ABA levels of high-CR ‘Zhuguang’ when endodormancy release, and then sharply increased and peaked during ecodormancy on January 23 (Figure 4A). The GA_3_ content of low CR ‘Hang Baishao’ declined to the lowest level when endodormancy release on January 09 and then increased gradually until late ecodormancy before again declining further. However, the GA_3_ content of high-CR ‘Zhuguang’ sharply decreased during endodormancy maintenance stage and then gradually decreased until February 27 (Figure 4A). Generally, the GA_3_ content in low CR ‘Hang Baishao’ was significantly higher than that in high-CR ‘Zhuguang’ throughout endodormancy and ecodormancy (Figure 4A and Supplementary Table 3). The ratio of GA_3_ to ABA decreased during endodormancy but increased during ecodormancy (Figure 4A).

The expression of the ABA synthesis-associated gene *PlNCED3* was upregulated during endodormancy maintenance stage (Supplementary Figure 1), while the GA synthesis-related gene *PlGA20ox8* was upregulated and *PlSPT* was downregulated in the two cultivars during endodormancy release (Figure 4B). In the ABA signal transduction pathway, *PlPYR* and *PlSNRK* were upregulated and *PlPP2C*, *PlABI5* and *PlbZIP* were downregulated in the two cultivars during endodormancy release (Figure 4B). The GA signal transduction-related genes *PlGID1* and *PlSPY* were both upregulated in the two cultivars during endodormancy release (Figure 4B). A heatmap of known genes involved in ABA and GA synthesis and signal transduction based on qRT–PCR was plotted (Supplementary Figure 1). Genes related to ABA signaling, such as *PlPP2C*, *PlABI5*, *PlSNRK*, and *PlbZIP*, displayed distinctly earlier expression in high-CR ‘Zhuguang,’ which corresponds to the early accumulated ABA content in contrast to that in low CR ‘Hang Baishao’ during endodormancy release (Figures 4A,B). Most GA signal transduction genes were first upregulated and then downregulated in both cultivars (Supplementary Figure 1). In addition, *PlXERICO*, which connects the ABA and GA signal transduction pathways, was obviously expressed earlier in high-CR ‘Zhuguang’ than in low CR ‘Hang Baishao’ (Figure 4B).

### Phylogenetic Tree and Gene Expression Analysis of Crucial MIKC*^C^*-Type MADS-Box Genes

**FIGURE 5:**
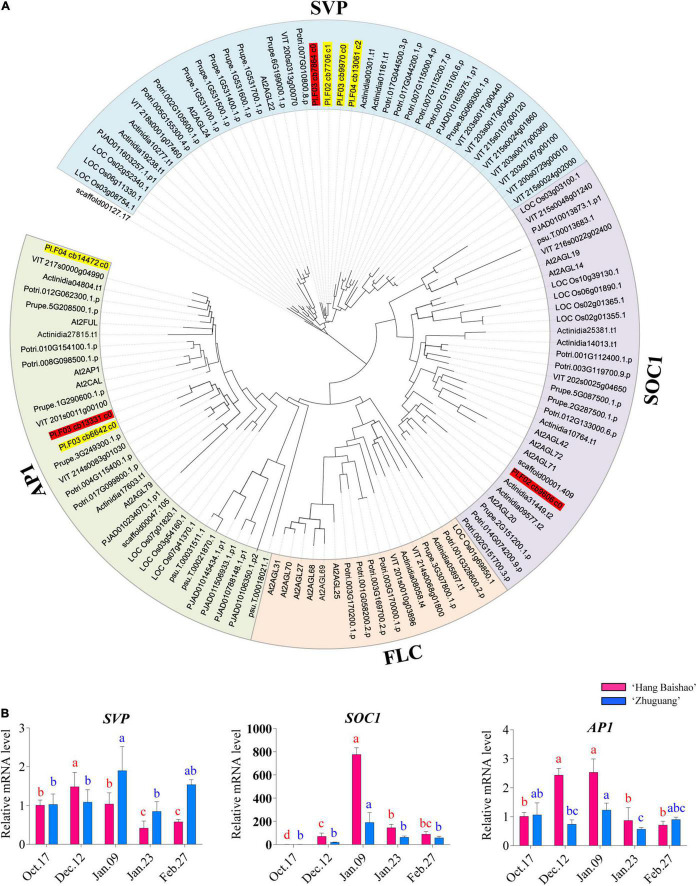
Phylogenetic analysis of the four MIKC*^C^*-type MADS-box genes in *P. lactiflora* with its homologs from the other plants and expression patterns of key MADS-box genes in the two cultivars during bud dormancy. **(A)** Phylogenetic analysis of MADS-box gene family in *P. lactiflora* and homologs from the other plants. The tree was generated after multiple sequence alignment using MAFFT and the ML method. The number at each node indicates the percentage of bootstrapping of a 1,000 replications. Scale bar = 0.05. The *P. lactiflora* MADS-box genes were marked with red and yellow colors, and the genes for qRT–PCR were marked with red color. The full names of the species were shown in the main body of the text. **(B)** Expression patterns of key MADS-box genes in the two cultivars during bud dormancy. Error bars represent standard deviation from three biological replicates and different letters indicate significant differences (*P* < 0.05). Differences were compared among different move dates for each cultivar.

A total of eight non-redundant unitranscripts were assigned in *P. lactiflora* (Supplementary Table 2) after searching the integrated transcriptome database with HMM profile of the MADS-box domain. All the genes from the nine species were phylogenetically classified into four clades (scaffolds were placed as the root branch), which included the AP1/FUL, SOC1, SVP, and FLC clades (Figure 5A). The eight transcripts in *P. lactiflora* contained four *SVP*, three *AP1* and one *SOC1* transcript. However, no *FLC* transcripts clustered in the FLC clade. Phylogenetic and sequence analysis of *SVP* transcripts showed only one *SVP* in *P. lactiflora*, while the other three resulted from alternative splicing events of the *SVP*. Similarly, one *AP1* and one *SOC1* were identified in *P. lactiflora* (Figure 5A).

The expression of the *PlSVP* was upregulated during early endodormancy release stage followed by a slight decrease until January 23 in the low-CR ‘Hang Baishao’ (Figure 5B). However, an increased expression of *PlSVP* in the high-CR ‘Zhuguang’ was observed before late endodormancy release, followed by downregulation when endodormancy release, which was later than that in low CR ‘Hang Baishao’ (Figure 5B). *PlSOC1* and *PlAP1*, which are downstream of *PlSVP*, were upregulated in low CR ‘Hang Baishao’ during endodormancy release. However, the expression of *PlSOC1* and *PlAP1* in the high-CR ‘Zhuguang’ was distinctly lower than that in the low-CR ‘Hang Baishao’ (Figure 5B and Supplementary Table 3).

### Changes in Physiological and Biochemical Indices and Gene Expression Related to Oxidative Stress

**FIGURE 6:**
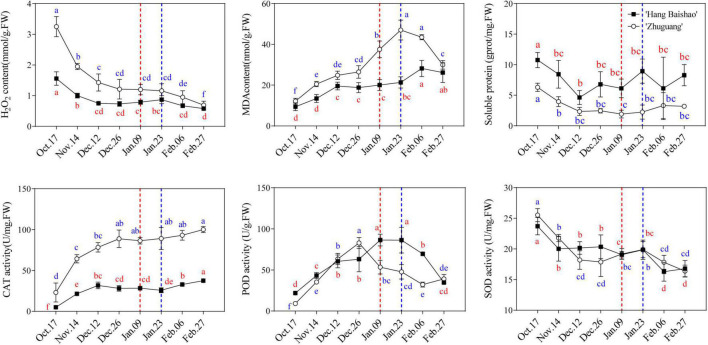
Effect of natural chilling on oxidative stress related physiological and biochemical indices in ‘Hang Baishao’ and ‘Zhuguang’ during bud dormancy. Dashed red line indicates the bud endodormancy release date of low CR ‘Hang Baishao’ and dashed blue line indicates the bud endodormancy release date of high-CR ‘Zhuguang.’ Error bars represent standard deviation from three biological replicates and different letters indicate significant differences (*P* < 0.05). Differences were compared among different move dates for each cultivar.

H_2_O_2_ decreased sharply before December 12 and gradually declined afterward, and the H_2_O_2_ content of the high-CR ‘Zhuguang’ was always higher than that of low CR ‘Hang Baishao,’ especially in the endodormancy maintenance and early endodormancy release stages (Figure 6 and Supplementary Table 3). In contrast, the MDA content gradually increased with the prolongation of natural chilling, and low CR ‘Hang Baishao’ reached its maximum value during ecodormancy, which was later than that for high-CR ‘Zhuguang’ when endodormancy release (Figure 6). Moreover, the MDA content of high-CR ‘Zhuguang’ was always higher than that of low CR ‘Hang Baishao’ (Figure 6 and Supplementary Table 3). The soluble protein content of both cultivars gradually decreased before December 12 and stabilized afterward (Figure 6). In general, the soluble protein content of low CR ‘Hang Baishao’ was always higher than that of high-CR ‘Zhuguang’ (Figure 6 and Supplementary Table 3). In contrast to the H_2_O_2_ content, the CAT activity gradually increased, and there were significant changes in CAT activity in the endodormancy maintenance BEM and early endodormancy release stages. However, the CAT activity of high-CR ‘Zhuguang’ was always higher than that of low CR ‘Hang Baishao’ (Figure 6 and Supplementary Table 3). Similarly, the activity of POD first increased and then decreased, and low CR ‘Hang Baishao’ reached its peak when endodormancy release, which was later than that of high-CR ‘Zhuguang’ which peaked in the middle endodormancy release stage. In contrast, the activity of SOD decreased rapidly during early endodormancy release stage in the two cultivars and then shows no significant change though trends downward after January 23 in both varieties (Figure 6).

**FIGURE 7:**
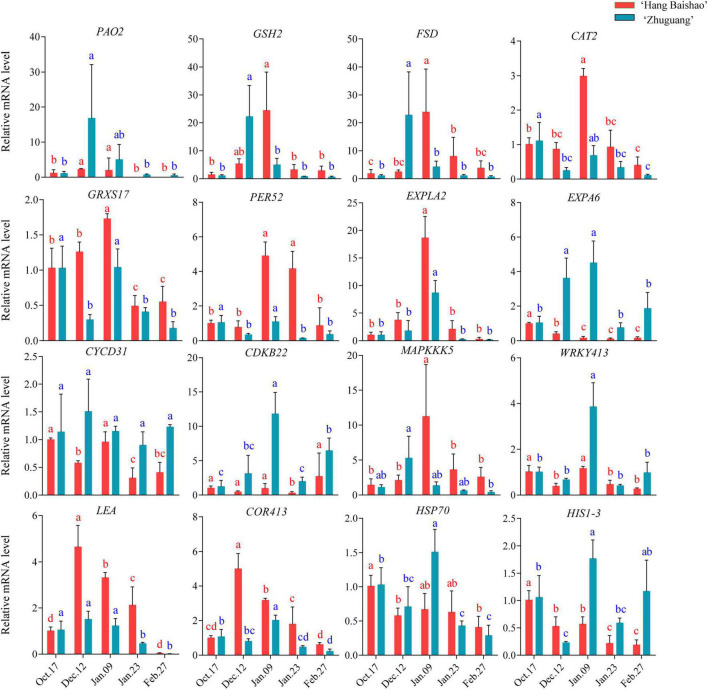
Effect of natural chilling on oxidative stress related genes expression in ‘Hang Baishao’ and ‘Zhuguang’ during bud dormancy. Error bars represent standard deviation from three biological replicates and different letters indicate significant differences (*P* < 0.05). Differences were compared among different move dates for each cultivar.

The expression of the ROS-generating gene *polyamine oxidase* 2 (*PlPAO2*) correlated with dormancy states. Notably, the expression of *PlPAO2* in the high-CR ‘Zhuguang’ was higher and earlier than in low CR ‘Hang Baishao’ during endodormancy release stage (Figure 7 and Supplementary Table 3). However, the upregulated ROS-scavenging genes (*PlGRXS17*, *PlPER52*, *PlGSH2*, and *PlCAT2*) were higher in low CR ‘Hang Baishao’ than in high-CR ‘Zhuguang’ during endodormancy release stage. Moreover, higher expression of stress-response genes (*PlWRKY413*, *PlCOR413*, *PlHSP70*, *PlHISI-3*, and *PlLEA*) and lower expression of cold response- and cell division-related genes (*PlMAPKKK5*, *PlLEA*, *PlCOR413*, and *PlEXLA2*) were observed in high-CR ‘Zhuguang’ than in low CR ‘Hang Baishao’ during endodormancy release stage (Figure 7 and Supplementary Table 3).

### Comparison of Histological and Cytological Observations Between the Two Cultivars

**FIGURE 8:**
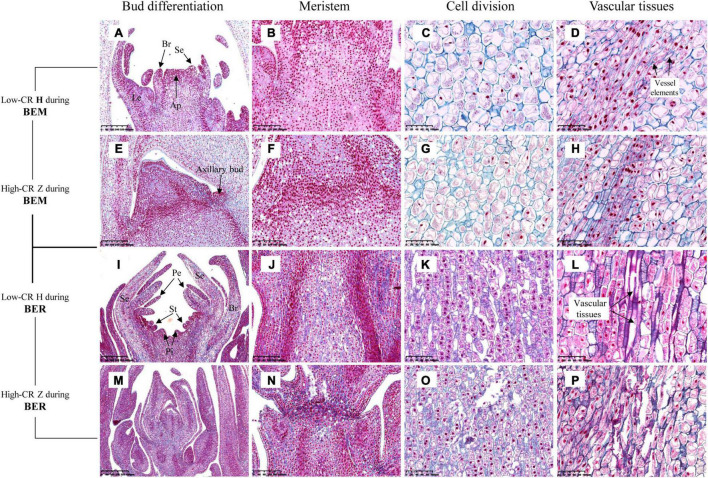
Histocytological observations of underground buds in the two cultivars during bud endodormancy release maintenance and release stages. **(A,E,I,M)** Differentiation of buds (scale bar: 400 μm). **(B,F,J,N)** Meristem inside the buds (scale bar: 200 μm). **(C,G,K,O)** Cell division (scale bar: 100 μm). **(D,H,L,P)** Vascular tissues (scale bar: 100 μm). H represents ‘Hang Baishao’; Z represents ‘Zhuguang.’ BEM, bud endodormancy maintenance; BER, bud endodormancy release.

To further confirm the relationship between ROS and bud endodormancy release, bud histology was observed in the crucial bud endodormancy release stage. The morphology of underground buds was observed on October 17 and January 09, which corresponded to the bud endodormancy maintenance and release stages, respectively (Figure 8). The results showed that the underground buds differentiated into Le, Se, and Br in the low-CR ‘Hang Baishao’ during endodormancy maintenance stage (Figure 8A). In addition, a large number of cells in the apical meristem were observed to divide, and some of them began to differentiate into vessel elements (Figures 8C,D). Compared with low CR ‘Hang Baishao,’ there was no obvious differentiation of buds during endodormancy maintenance stage, and the division of apical meristem cells was relatively lower in the high-CR ‘Zhuguang’ (Figures 8E,G,H). Notably, most of the cell division did not occur in the apical meristem but occurred below it and near the axillary bud (Figure 8F). The complete floral organs of low CR ‘Hang Baishao’ were differentiated, and complete vascular tissues were formed due to vigorous cell division and differentiation during late endodormancy release stage (Figures 8I–L). In contrast, the buds in the high-CR ‘Zhuguang’ failed to differentiate and showed abortion associated with ruptured cells and blocked vascular tissues (Figures 8M–P).

### Multiple Index Correlation Analysis

The correlations between morphological indices and physiological indices were analyzed using Spearman’s correlation analysis and displayed with heatmaps (Table 4). Generally, the correlation coefficients obtained for these indices corresponding to carbohydrate metabolism, plant hormones and the ROS system in low CR ‘Hang Baishao’ were higher than those in high-CR ‘Zhuguang,’ especially the bud break and growth related morphological indices BPF, WFS, and ANS, which were considered closely related to bud dormancy progress as noted by their darker color/significance in the heatmap (Table 4).

**TABLE 4:** Correlation analysis of bud endodormancy release related crucial indices in the low-CR ‘Hang Baishao’ and high-CR ‘Zhuguang.’

H	ZR	IAA	ABA	GA3	GA3/ABA	BR	JAMe	SUC	SUG	STA	POD	SOD	CAT	MDA	H2O2	BPF	WFS	ANS
ZR	1.00																	
IAA	−0.39	1.00																
ABA	−0.70**	0.46	1.00															
GA3	0.83**	−0.58*	−0.64*	1.00														
GA3/ABA	0.88**	−0.56*	−0.88**	0.90**	1.00													
BR	0.11	0.17	−0.23	0.36	0.30	1.00												
JAMe	−0.14	0.01	0.02	0.27	0.05	0.63*	1.00											
SUC	−0.64**	0.44	0.80**	−0.70**	−0.81**	−0.47	−0.19	1.00										
SUG	−0.69**	0.44	0.78**	−0.78**	−0.84**	−0.55*	−0.23	0.95**	1.00									
STA	0.60*	−0.15	−0.75**	0.52*	0.73**	0.35	0.07	−0.46	−0.50	1.00								
POD	−0.87**	0.50	0.89**	−0.80**	−0.94**	−0.26	0.04	0.80**	0.83**	−0.74**	1.00							
SOD	0.23	0.11	−0.50	0.20	0.29	0.10	0.09	−0.26	−0.29	0.30	−0.26	1.00						
CAT	−0.81**	0.54*	0.59*	−0.68**	−0.68**	0.01	0.11	0.59*	0.65**	−0.35	0.73**	−0.23	1.00					
MDA	−0.84**	0.47	0.90**	−0.74**	−0.92**	−0.29	0.02	0.87**	0.85**	−0.71**	0.92**	−0.31	0.72**	1.00				
H2O2	0.68**	−0.70**	−0.59*	0.71**	0.66**	−0.13	0.05	−0.54*	−0.60*	0.25	−0.64*	0.38	−0.84**	−0.60*	1.00			
BPF	−0.71**	0.55*	0.82**	−0.83**	−0.87**	−0.39	−0.32	0.91**	0.94**	−0.53*	0.84**	−0.36	0.70**	0.86**	−0.75**	1.00		
WFS	0.93**	−0.34	−0.93**	0.87**	0.96**	0.51	0.10	−0.87**	−0.92**	0.86**	−0.93**	0.59	−0.73*	−0.96**	0.59	−0.89**	1.00	
ANS	−0.42	0.37	0.62*	−0.62*	−0.66**	−0.58*	−0.38	0.83**	0.79**	−0.42	0.69**	−0.07	0.32	0.62*	−0.35	0.80**	−0.74**	1.00

**Z**	**ZR**	**IAA**	**ABA**	**GA3**	**GA3/ABA**	**BR**	**JAMe**	**SUC**	**SUG**	**STA**	**POD**	**SOD**	**CAT**	**MDA**	**H2O2**	**BPF**	**WFS**	**ANS**

ZR	1.00																	
IAA	0.81**	1.00																
ABA	−0.30	−0.17	1.00															
GA3	0.77**	0.60*	−0.23	1.00														
GA3/ABA	0.58*	0.29	−0.66**	0.77**	1.00													
BR	0.25	0.42	−0.24	0.25	0.04	1.00												
JAMe	−0.45	−0.10	0.38	−0.74**	−0.75**	0.15	1.00											
SUC	−0.01	0.18	−0.75**	−0.09	0.25	0.37	0.08	1.00										
SUG	−0.15	−0.04	−0.65**	−0.20	0.24	0.23	0.13	0.92**	1.00									
STA	0.42	0.46	0.06	0.46	0.25	−0.15	−0.29	−0.27	−0.44	1.00								
POD	−0.58*	−0.32	0.58*	−0.81**	−0.93**	0.05	0.83**	−0.18	−0.12	−0.33	1.00							
SOD	0.54*	0.45	−0.48	0.74**	0.69**	−0.04	−0.79**	0.25	0.07	0.49	−0.83**	1.00						
CAT	−0.67**	−0.57*	0.28	−0.89**	−0.69**	−0.16	0.76**	−0.02	0.07	−0.54*	0.78**	−0.85**	1.00					
MDA	−0.78**	−0.64**	0.32	−0.82**	−0.72**	−0.01	0.63*	0.00	0.13	−0.40	0.77**	−0.65**	0.657**	1.00				
H2O2	0.70**	0.61*	−0.30	40.82**	0.59*	0.17	−0.67**	0.04	−0.13	0.64**	−0.71**	0.89**	−0.91**	−0.62*	1.00			
BPF	−0.63*	−0.56*	−0.13	−0.86**	−0.41	−0.28	0.53*	0.38	0.52*	−0.58*	0.51	−0.55*	0.79**	0.58*	−0.77**	1.00		
WFS	−0.02	0.03	−0.08	−0.15	−0.07	−0.06	0.29	0.14	0.12	−0.10	−0.05	−0.20	0.35	−0.13	−0.25	0.29	1.00	
ANS	−0.44	−0.37	−0.17	−0.75**	−0.33	−0.05	0.59*	0.43	0.51	−0.48	0.54*	−0.52*	0.66**	0.62*	−0.63*	0.83**	0.06	1.00

The symbol “**” indicates extreme significance (P < 0.01), and the symbol “*” represents significance (P < 0.05). The darker the background color is, the more significant the correlation is. Green indicates a positive correlation, and red indicates a negative correlation. H, ‘Hang Baishao’; Z, ‘Zhuguang’; ZR, zeatin riboside; IAA, indole-3-acetic acid; BR, brassinosteroid; JAMe, methyl jasmonate; SUC, sucrose; SUG, sugars; STA, starch. The other full names of the acronyms are shown in the main body of the article. The contents of IAA, ZR, BR and JAMe were shown in Supplementary Figure 2.

Compared with ZR, IAA, and BR, ABA, and GA_3_ had higher correlation coefficients in both low CR ‘Hang Baishao’ and high-CR ‘Zhuguang,’ but these coefficients were still lower than those for GA_3_/ABA (Table 4). Correlation analysis among carbohydrate metabolism indices showed significantly higher correlation coefficients in low CR ‘Hang Baishao’ than in high-CR ‘Zhuguang.’ In addition, ROS system-related indices both had high correlation coefficients in the two cultivars, especially H_2_O_2_, CAT, and POD (Table 4).

## Discussion

### Knowing the Underlying Mechanism in Bud Endodormancy Release Is Vital for Widely Planting Herbaceous Peony in Warm Winter Climates

Herbaceous peony is mainly cultivated in temperate regions with relatively cool climate of the Northern Hemisphere, including North America, Europe, and Asia, the introduction of herbaceous peony from higher to lower latitudes can enhance the ornamental diversity and promote plant landscaping in low-latitude areas (N 30°00′–S 30°00′ areas) (Zhang et al., 2019b; Wang et al., 2020). However, the global warming trend aggravates the lack of chilling fulfillment at low latitudes or beyond the natural habitat of herbaceous peony, and seriously hinders bud endodormancy release, results in low flowering and thus degrades the growth of plants year by year (Zhang et al., 2017; Baumgarten et al., 2021). Introducing, screening and breeding cultivars is not enough to solve the problem. However, studying CR traits and knowing the underlying mechanism in the obstacles to bud endodormancy release may provide effective ways to solve the CR deficiency caused by the warm winter climate (Wang et al., 2020).

The length of the endodormancy period is dependent on the CRs of each species, subspecies or cultivars (Prudencio et al., 2020). How to shorten the duration of the endodormancy period of buds and make them easier to release (showing the low-CR trait) needs to be studied. This problem can be further explored from the aspects of carbohydrate metabolism, ROS signaling, hormone regulation and the interaction of the above factors. In addition, the mining of related key genes is of great help for elucidating the mechanism of bud dormancy and obtaining transgenic plants with a short endodormancy period, which could greatly accelerate the breeding of germplasms with a low-CR trait (Zhang et al., 2017). This method is fundamentally different from the introduction and cultivation of low-CR cultivars or forcing cultivation to solve the obstacles to bud endodormancy release caused by warm winters. Many similar researches have also been conducted for deciduous fruit-bearing, forest and tea trees as well as tree peony (Zhang et al., 2015; Azizi Gannouni et al., 2017; Xue et al., 2018; Gougherty et al., 2021). These researches are vital for the introduction, planting and production of economic plants at low latitudes under warm winter climates.

### Sugars Actively Participate in the Bud Dormancy Transition of Herbaceous Peony

Many studies have shown that sugar is closely related to dormancy (Chao and Serpe, 2010; Rubio et al., 2019), the levels of which were confirmed to be correlated with the transition of vegetative buds from paradormancy to endodormancy (Kaufmann and Blanke, 2017). Although less studied than it is in terminal vegetative and floral buds, dormancy-associated conversion of starch to sucrose occurs in other underground buds from other herbaceous plants (Anderson et al., 2005). In this study, it was found that sugar levels were associated with the dormancy process. In both cultivars, sugar levels decreased slightly during the endodormancy maintenance stage; rise and peaked during endodormancy; and then they declined during ecodormancy (Figure 2A). The continuous conversion of starch to sugars has been correlated with the transition of the bud dormancy stage. Thus, carbohydrates in bud endodormancy release could serve as a marker for the division of dormancy stages (Kaufmann and Blanke, 2017). Interestingly, *SUCROSE TRANSPORTER 2* (*SUC2*) which encodes a sucrose transport protein, up-regulated during endodormancy release stage and down-regulated after endodormancy release both in the two cultivars (Supplementary Figure 2). However, the expression level of *SUC2* in the low-CR ‘Hang Baishao’ was always higher than in the high-CR ‘Zhuguang’ and correlated with sugar levels, which indicated that accumulation and transportation of sugars might play important roles in the regulation of endodormancy release (Figure 2A and Supplementary Figure 2). In addition, the results showed that sucrose was the most abundant soluble sugar; the content of sucrose significantly increased during endodormancy release stage (Figure 2A and Supplementary Figure 2), which suggested that sucrose may be used to provide energy following the release of bud endodormancy (Mornya and Cheng, 2011).

Several reports have shown that low temperatures induce starch degradation, and the main enzymes involved in this pathway, including AMY (α-Amylase), BMY (β-Amylase), SUS (Sucrose synthase), and SPS (Sucrose phosphate synthase), are differentially regulated and facilitate dormancy release (Chao and Serpe, 2010; Liu X. et al., 2020). For instance, *BMY* has been identified as being regulated by *The C – repeat binding factor* (*CBF*) gene which is a key regulator of cold-acclimation in many plants (Li W. et al., 2020). In this study, *PlAMY* and *PlBMY* were both upregulated until endodormancy release in the two cultivars (Figure 2B). Similarly, *PlSUS* and *PlSPS*, which are related to sucrose synthesis, were upregulated during endodormancy release stage (Figure 2B). Nevertheless, the expression of *PlAMY*, *PlSPS*, and *PlSUS* was lower in the high-CR ‘Zhuguang’ and corresponded with a lower content of soluble sugars (Figures 2A,B and Supplementary Table 3). This suggested that the low-CR ‘Hang Baishao’ could promote bud endodormancy release through higher expression of sucrose synthesis genes and starch decomposition genes. Although we cannot rule out that the lower sucrose content in high-CR ‘Zhuguang’ is not because it initiated the conversion of starch to sugars later in the fall and thus lagged behind low CR ‘Hang Baishao’ or because it had accumulated less starch than low CR ‘Hang Baishao’ during the growing season. Some accumulated sugars can act as signals to trigger a series of transcription programs to promote bud endodormancy release through its regulators, such as SnRK1, to regulate the expression of certain cold-related genes and could mediate ABA and/or ethylene synthesis to respond to cold temperatures (Rubio et al., 2019; Xue et al., 2021). SnRK1 and ABA both negatively regulate the TARGET OF RAPAMYCIN (TOR) kinase complex. Repression of the TOR kinase inhibits growth in all Eucaryotes that have been investigated. This complex serves as the central hub for collecting information on nutrients and stress and transducing that information to control cell division and growth (Burkart and Brandizzi, 2020), and thus may well play a central role in controlling bud dormancy.

### The Balance Between Abscisic Acid and Gibberellin Regulates the Bud Dormancy of Herbaceous Peony

The importance of hormone homeostasis in bud dormancy has been well reviewed (Wang et al., 2015; Falavigna et al., 2018). In many cases, the determination of dormancy or sprouting mainly depends on an intrinsic balance of GA and ABA biosynthesis and catabolism (Yang et al., 2021). GA positively regulates the release of dormancy, whereas ABA is involved in the induction and maintenance of dormancy (Horvath et al., 2003). ABA levels may increase with short days and low temperatures in autumn, resulting in the inhibition of cell division and the induction of endodormancy (Polanski et al., 2020). The findings in this study regarding endogenous GA/ABA confirmed the decreasing trend during endodormancy and increasing trend during ecodormancy in both cultivars (Figure 4A), which is consistent with results in grapes, sweet cherry and tree peony (Sudawan et al., 2016; Falavigna et al., 2018; Xue et al., 2018). After comparing the ratio of GA/ABA in the two cultivars, it was found that the GA/ABA of the low-CR ‘Hang Baishao’ was higher than that of high-CR ‘Zhuguang’ (Figure 4A and Supplementary Table 3) prior to bud endodormancy release, indicating it may play a role in endodormancy maintenance and could possibly be responsible for a higher level of dormancy establishment in high-CR ‘Zhuguang.’ However, GA/ABA were at similar levels at the point of endodormancy release and thereafter, indicating that the GA/ABA ratio may not play a major role in endodormancy release in peony.

However, it should be noted that the spike in ABA levels in low CR ‘Hang Baishao’ corresponded to a brief warming trend near the end of January. ABA also negatively regulates the TOR kinase complex (Burkart and Brandizzi, 2020). This observation suggests that perhaps, this increase in ABA levels might be a protective mechanism that has evolved in low CR ‘Hang Baishao’ to prevent growth and protect it from such events, as they would be more common in the warmer climates to which low CR ‘Hang Baishao’ is adapted. It should also be noted that a similar if less dramatic trend was observed for MDA levels suggesting some correlative mechanism- either one regulating the other or both being regulated by some other factor.

Abscisic acid levels are positively regulated by the DELLA protein by upregulating the expression of the *PlXERICO* gene (Guan et al., 2019). In this study, we observed upregulation of *PlXERICO* near the endodormancy release in the two cultivars (Figure 4B). Interestingly, *PlXERICO, PlSNRK*, and *PlPP2C* were highly coordinately expressed, and roughly correlated with ABA levels, but only in low CR ‘Hang Baishao.’ These correlations do not hold for high-CR ‘Zhuguang,’ and thus may point to another possible adaptation to warmer winters in low CR ‘Hang Baishao.’ Regardless of the actual ABA levels, *PlXERICO* was slightly upregulated near the end of endodormancy maintenance in the high-CR ‘Zhuguang,’ which was about 2 weeks earlier than in low CR ‘Hang Baishao’ (Figure 4B). These observations suggest other factors may play a role in ABA accumulation and endodormancy release, particularly in high-CR ‘Zhuguang.’ Interestingly, contrary to ABA levels, GA levels and associated gene expression appears to correspond to the timing of endodormancy release in both cultivars, although it tends to precede it by at least 2 weeks. This would be consistent with the observed role of GA in opening symplastic connections prior to and facilitating bud endodormancy release in other systems (Rinne et al., 2011), but inconsistent with an inhibitory role for ABA in the same process (Tylewicz et al., 2018).

Gibberellin concentrations were found to increase during the dormancy phase transition in low CR ‘Hang Baishao’ but not in high-CR ‘Zhuguang’ (Figure 4). A similar situation was implicated in peach and apicot with low-CR genotypes having higher GA_3_ levels than the high-CR genotype (Yu et al., 2020). Increased active GA further controls bud dormancy and flowering by promoting the expression of *PlSOC1*. In tree peony, altered GA levels affected the expression of *SVP* and *SOC1* associated with flowering time and improved flower quality (Guan et al., 2019). However, in this study, changes in GA levels do not correspond to changes in *PlSOC1* and *PlSVP* expression, as these genes do not show differences in expression between the two cultivars until January 09, while GA levels are significantly different starting November 14. That said, *PlSOC1* and *PlSVP* expression does correlate to the differences in bud endodormancy release between the two cultivars (Figure 5B and Supplementary Table 3) as would be expected, with significantly higher expression of *PlSVP* and significantly lower *PlSOC1* levels in high-CR ‘Zhuguang’ corresponding to bud endodormancy release.

### Reactive Oxygen Species Over-Accumulated to Increase Resistance to Warm Winters in the High-Chilling Requirement Cultivar of Herbaceous Peony During Bud Endodormancy Release

Warm winters will not only lead to obstacles to bud endodormancy release but are also accompanied by a series of abiotic stresses. ROS is over-accumulated under abiotic stresses, which can cause oxidative damage to cell structures, leading to restriction of plant growth or even to death (Choudhury et al., 2017; Ishibashi et al., 2017) and must be strictly controlled (Barba-Espin et al., 2011; Sudawan et al., 2016). Antioxidant control systems involving the enzymes CAT, SOD, and POD are increased to maintain the level of ROS in plant tissues (Mujahid et al., 2020). The high ROS level caused the upregulation of *PlGRXS17*, *PlCAT2*, and *PlPER52*, which led to increased activities of CAT and POD in both ‘Hang Baishao’ and ‘Zhuguang’ during endodormancy maintenance stage (Figures 6, 7). However, the expression levels of these ROS scavenging genes were lower in high-CR ‘Zhuguang’ (Figure 7 and Supplementary Table 3), which suggests it is under higher oxidative stress. Yet, it must be recognized that CAT activity was still higher in high-CR ‘Zhuguang.’ This result indicates that it was difficult for the ROS scavenging system of high-CR ‘Zhuguang’ to maintain ROS at a normal level, which could be one of the factors that induced obstacles to bud endodormancy release (Porcher et al., 2020). It should be noted that the highest levels of damage, as indicated by MDA concentrations, was observed in high-CR ‘Zhuguang’ during one of the unusually warm periods in late January (Figure 6). Thus, suggesting the hypothesis that high-CR ‘Zhuguang’ incurs greater damage than low CR ‘Hang Baishao’ during short warming periods in winter.

High levels of H_2_O_2_ early in endodormancy fell precipitously and then stabilized slightly earlier in low CR ‘Hang Baishao’ than in high-CR ‘Zhuguang.’ Also, the levels of H_2_O_2_ was higher in high-CR ‘Zhuguang’ than in low CR ‘Hang Baishao’ and roughly negatively correlated with expression of the GA synthesis gene *PlGA20ox8* and positively correlated with expression of GA synthesis suppressor gene *PlSPT* in the two cultivars (Figures 4B, 6 and Supplementary Table 3). These observations would be consistent with oxidative stress stabilizing earlier in low CR ‘Hang Baishao’ and opening of the symplastic channels earlier in low CR ‘Hang Baishao’ than in high-CR ‘Zhuguang.’ The coordination of the H_2_O_2_ levels with GA synthesis and the stabilization of H_2_O_2_ levels just prior to increases in *PlGA20ox8* expression opens the intriguing possibility that H_2_O_2_ might regulate GA synthesis and thus be a driver for bud endodormancy release and provides a second way for oxidative stress levels to regulate bud endodormancy release in peony.

Interestingly, in grapes, higher ROS production has been hypothesized to serve as a signal to enhance endodormancy release (Halaly et al., 2008). Contrary to that hypothesis, in this study, high levels of H_2_O_2_ and higher expression of *PlWRKY413*, *PlHSP70*, and *PlbZIP* were found in the high-CR ‘Zhuguang’ (Figure 7 and Supplementary Table 3). This further indicated that high-CR ‘Zhuguang’ showed strong oxidative stress during endodormancy release stage, but that it did not lead to bud break.

### A Proposed Model for the Different Formation Mechanisms of Bud Endodormancy Release in Herbaceous Peony Cultivars With High- and Low-Chilling Requirement Traits Under Warm Winters

**FIGURE 9:**
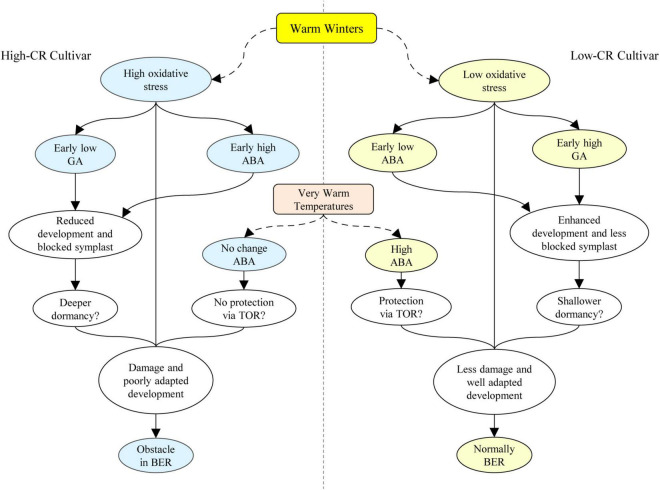
Proposed mechanistic model for the obstacle in bud dormancy release caused by warm winters in herbaceous peony. BER, bud endodormancy release.

Through a detailed comparative study on herbaceous peonies with high- and low- CR traits during bud endodormancy release, an important role for ROS and GA, and a potential role for ABA in protecting southern-adapted cultivars like ‘Hang Baishao’ from damage during warm spells- although the mechanism is unclear, it is possible that it acts through ABA repression of *TOR*. In the high-CR cultivar ‘Zhuguang,’ the insufficient chill accumulation caused by warm winters results in high level of ROS during the early endodormancy maintenance period, and a high level of ROS subsequently correlates with an earlier accumulation of ABA in high-CR ‘Zhuguang’ than in low CR ‘Hang Baishao.’ High oxidative stress in high-CR ‘Zhuguang’ also correlates to cellular damage and thus poorer adaptation to warmer winters. Lower GA levels in high-CR ‘Zhuguang’ may lead to blocked vascular tissue and abnormal bud differentiation potentially by inhibiting the expression of *PlSOC1*, which finally create obstacles to bud endodormancy release under warm winters (Figure 9).

## Data Availability Statement

The original contributions presented in the study are included in the article/Supplementary Material, further inquiries can be directed to the corresponding authors.

## Author Contributions

XW, JZ, and YX conceived and designed the entire study plan. XW completed most of the experiments. RZ, QH, and XS completed the partial experiments. XW, DL, JZ, and DH analyzed the data and drafted the manuscript. JZ, DH, DL, and YX reviewed the manuscript. All authors approved the final manuscript.

## Conflict of Interest

The authors declare that the research was conducted in the absence of any commercial or financial relationships that could be construed as a potential conflict of interest.

## Publisher’s Note

All claims expressed in this article are solely those of the authors and do not necessarily represent those of their affiliated organizations, or those of the publisher, the editors and the reviewers. Any product that may be evaluated in this article, or claim that may be made by its manufacturer, is not guaranteed or endorsed by the publisher.

## Footnotes

1
http://pfam.xfam.org/
