# Supplementary material for: Comparative Study on Physiological Responses and Gene Expression of Bud Endodormancy Release Between Two Herbaceous Peony Cultivars (Paeonia lactiflora Pall.) With Contrasting Chilling Requirements
Source: Front Plant Sci. 2022 Feb 2;12:772285. doi: 10.3389/fpls.2021.772285 (PMC8847303; doi:10.3389/fpls.2021.772285)
Supplement: Supplementary file 4 [file Data_Sheet_1.PDF]

Supplementary File S1 | The quality and quantity of RNA and PCR amplification curves and melt curves in this study

1 Total RNA concentration

Cultivar	Dates	Biological replicates	Nucleic acid (ng/μL)	260/280	260/230
‘Hang Baishao’	Oct. 17	h 1-1	421.20	2.10	2.20
		h 1-2	448.10	2.12	1.50
		h 1-3	264.40	2.15	2.16
	Dec. 12	h 2-1	418.00	2.13	1.84
		h 2-2	601.40	2.14	2.22
		h 2-3	625.40	2.13	0.98
	Jan. 09	h 3-1	326.80	2.16	0.82
		h 3-2	402.60	2.12	2.20
		h 3-3	340.10	2.14	1.62
	Jan. 23	h 4-1	441.80	2.09	2.15
		h 4-2	400.70	2.13	1.22
		h 4-3	487.00	2.08	2.01
	Feb. 27	h 5-1	939.90	2.13	2.35
		h 5-2	868.80	2.14	2.37
		h 5-3	934.50	2.14	2.00
‘Zhuguang’	Oct. 17	z 1-1	121.60	2.06	1.37
		z 1-2	280.10	2.13	1.13
		z 1-3	131.90	2.07	1.22
	Dec. 12	z 2-1	281.00	2.15	0.95
		z 2-2	364.00	2.14	1.32
		z 2-3	552.70	2.14	2.22
	Jan. 09	z 3-1	566.80	2.17	1.95
		z 3-2	716.70	2.17	2.01
		z 3-3	891.70	2.17	1.46
	Jan. 23	z 4-1	887.50	2.16	1.42
		z 4-2	702.90	2.13	2.22
		z 4-3	652.00	2.14	1.49
	Feb. 27	z 5-1	867.50	1.95	1.25
		z 5-2	1014.20	2.15	2.06
		z 5-2	738.60	2.16	1.99

2 Integrity of RNA in the buds of ‘Hang Baishao’ and ‘Zhuguang’

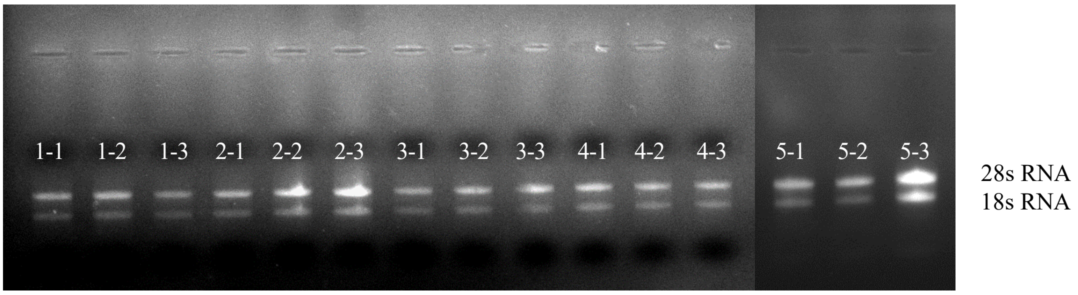

Figure. 1 | RNA extraction in the buds of ‘Hang Baishao’**.** RNA ladders from left to right represent three biological replicates of the five move dates, respectively.

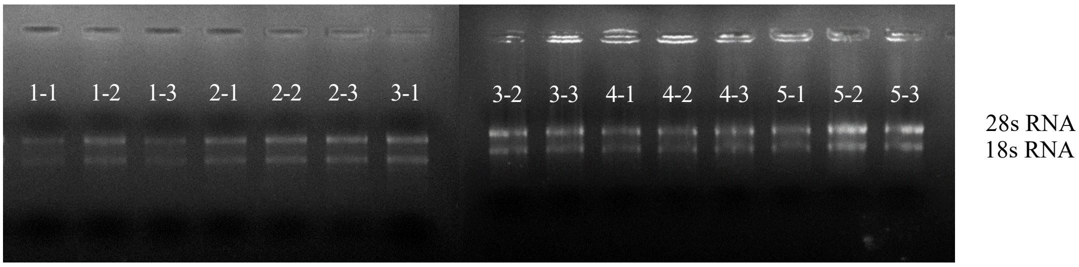

Figure. 2 | RNA extraction in the buds of ‘Zhuguang’. RNA ladders from left to right represent three biological replicates of the five move dates, respectively.

3 The validity of the reference gene *ATUBA*

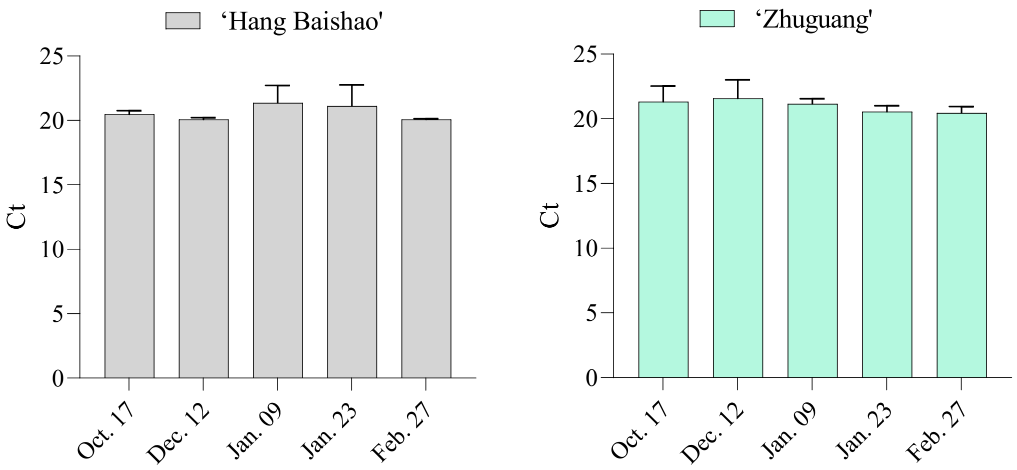

Figure. 3 | Threshold Cycles (Cts) of *ATUBA* in the buds of ‘Hang Baishao’ and ‘Zhuguang’ during the winter of 2018-2019. Values are means ± standard deviation (SD) of three biological replicates.

4 PCR amplification curves and melt curves of each pair of primer in this study.

PP2C

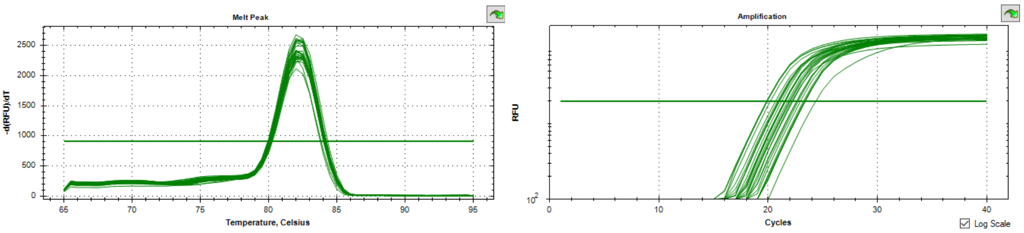

ABI5

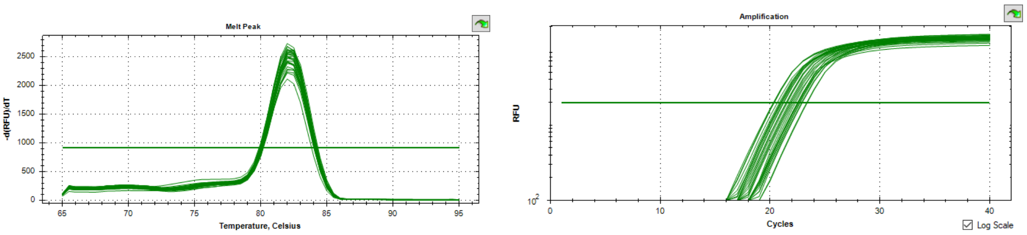

ABF3

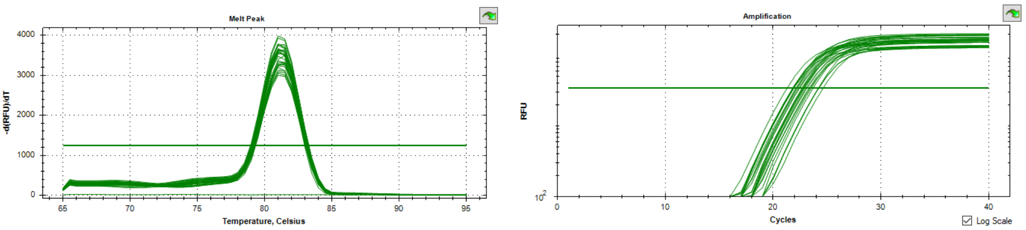

NCED3

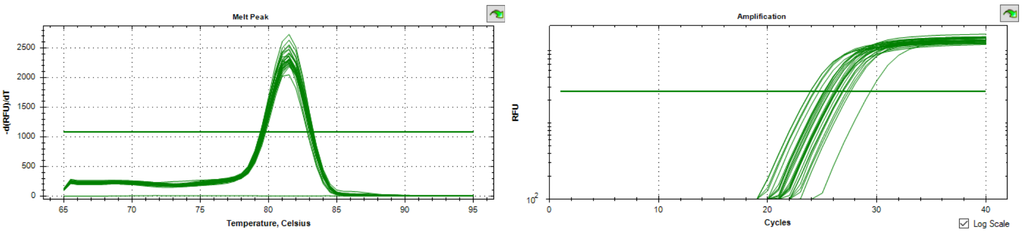

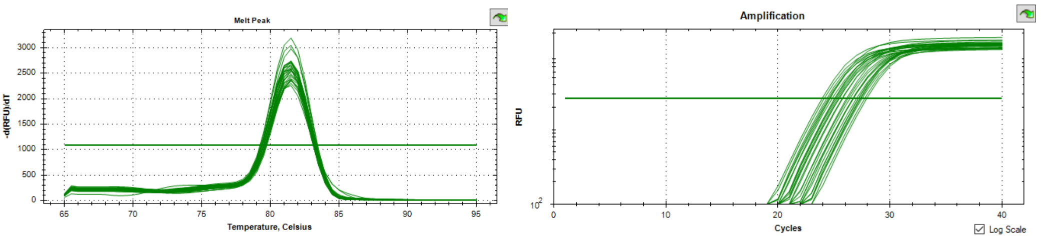

NCED4

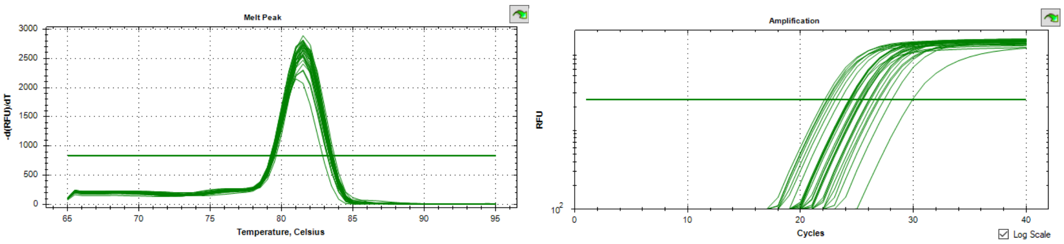

bZIP

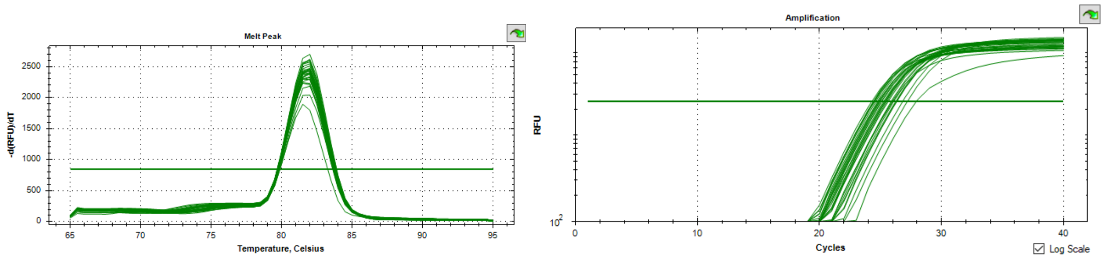

PYR

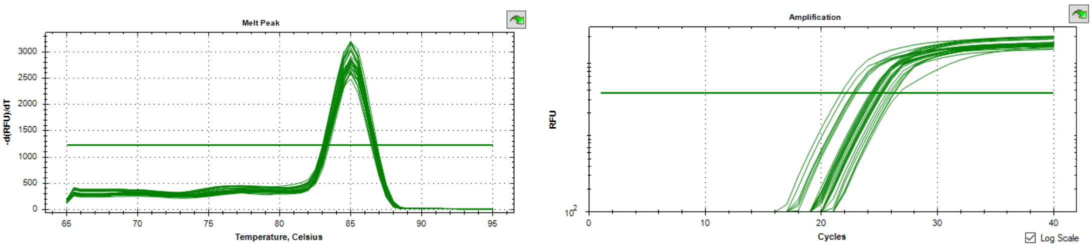

SOC1

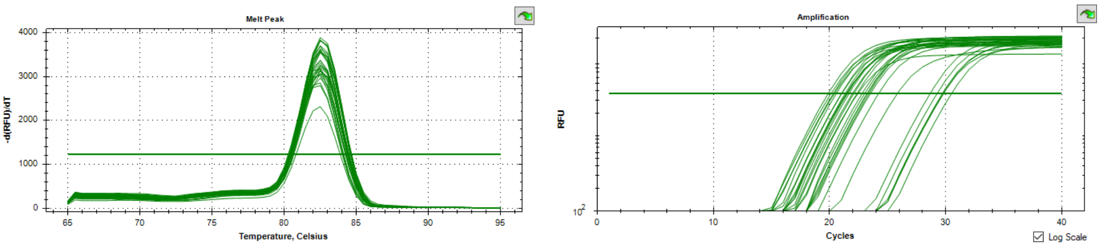

SVP

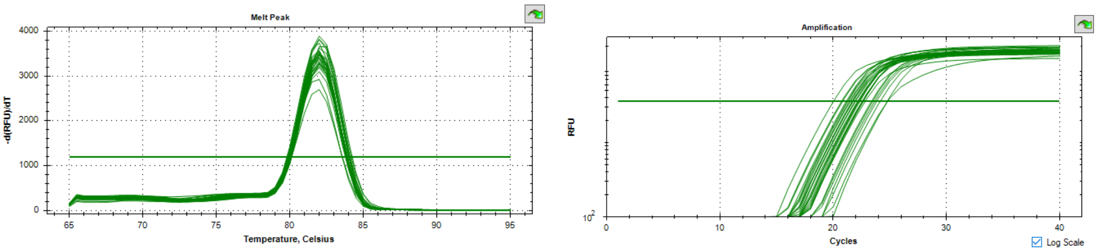

AP1

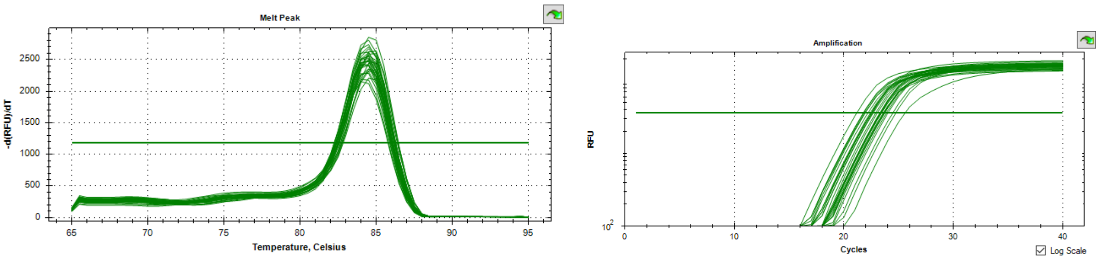

SPL

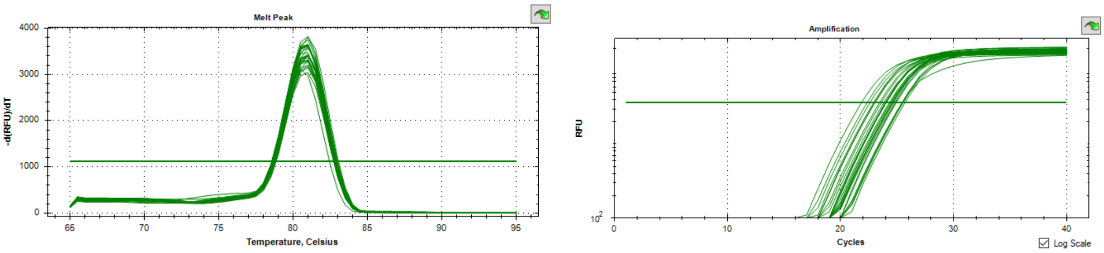

CBF4

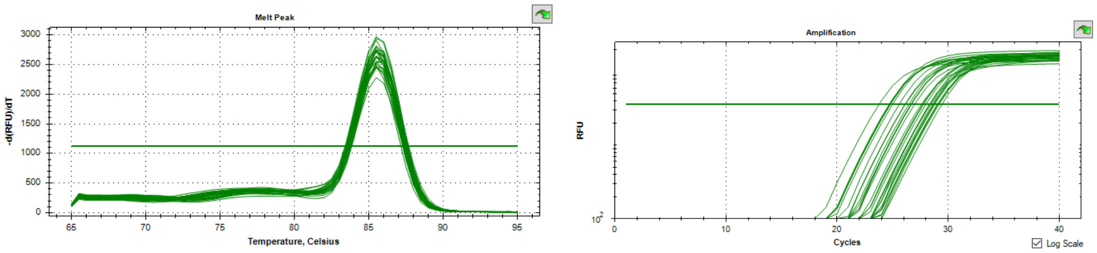

SS3

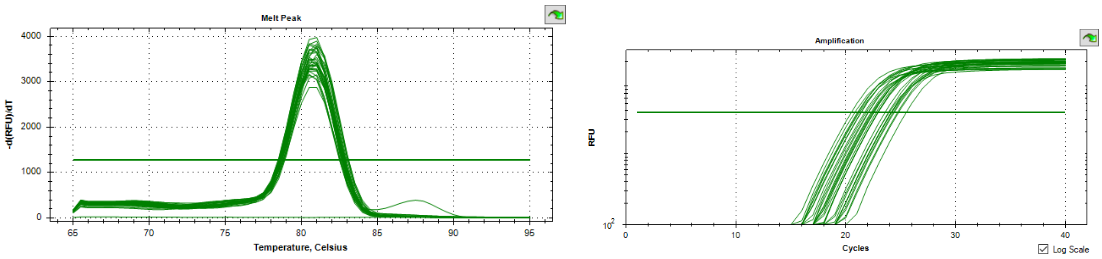

BMY

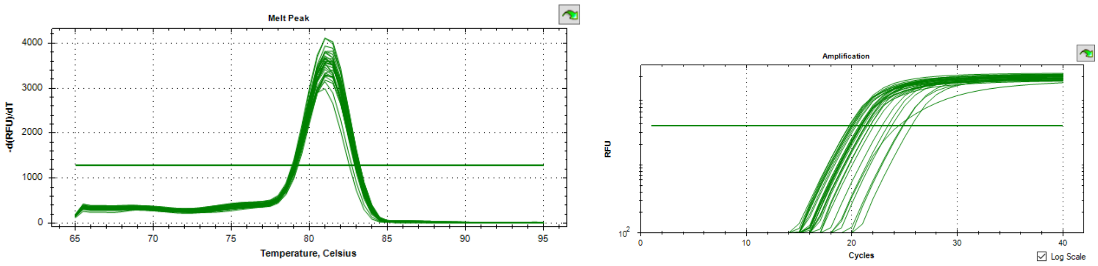

CWINV

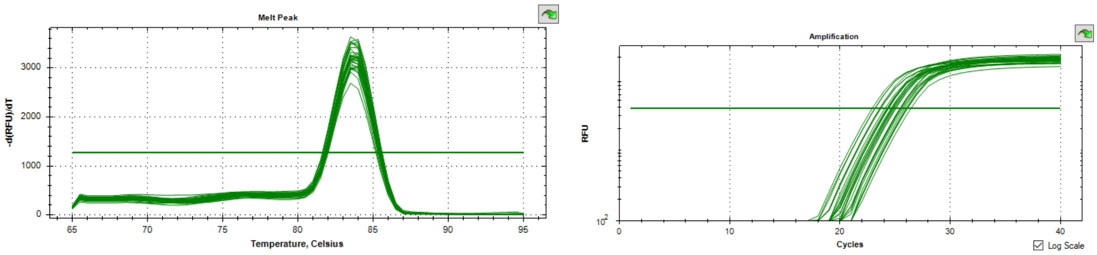

SUS

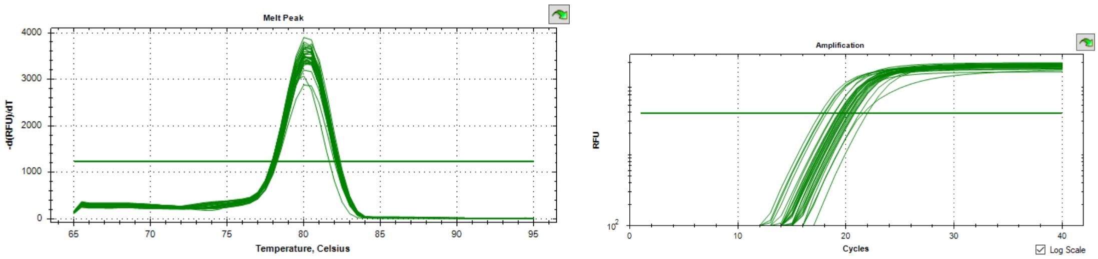

APL

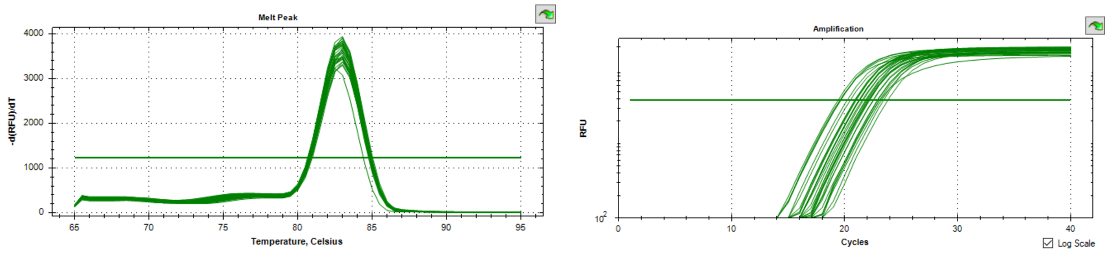

SPS

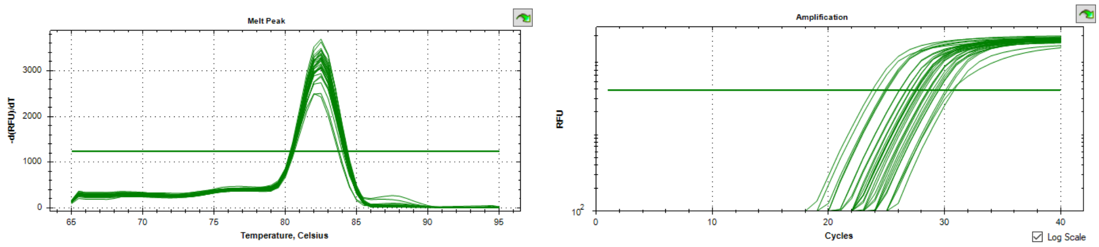

PFK3

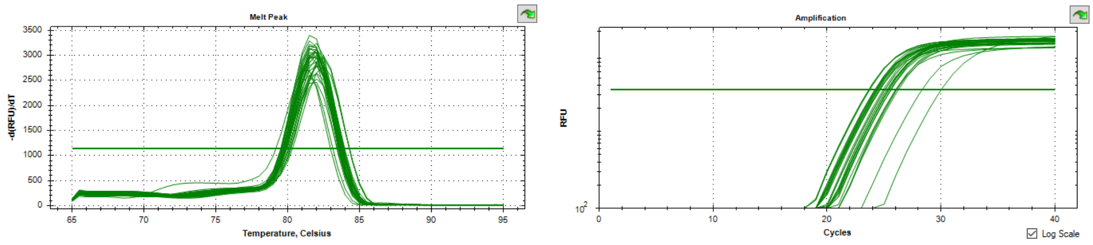

BGLU

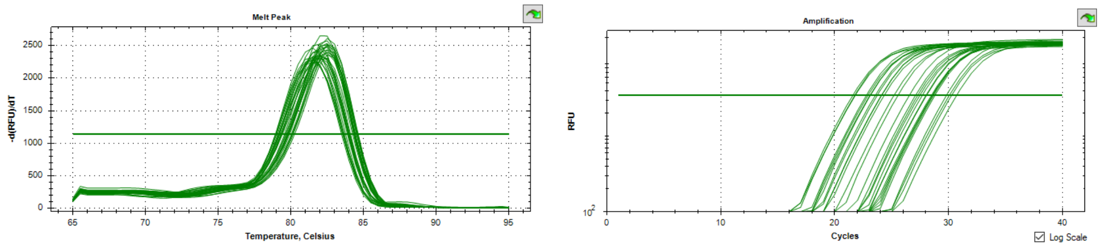

AMY

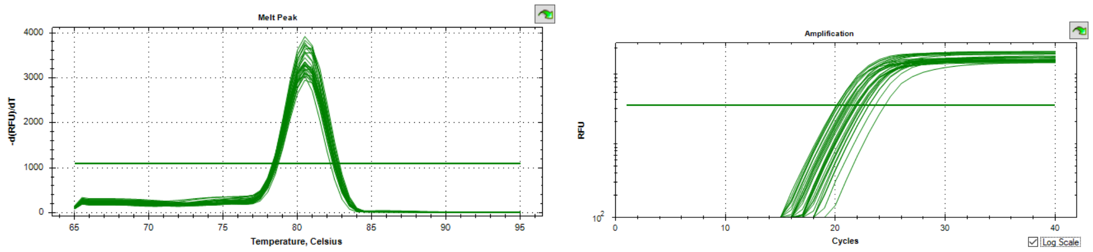

SPT

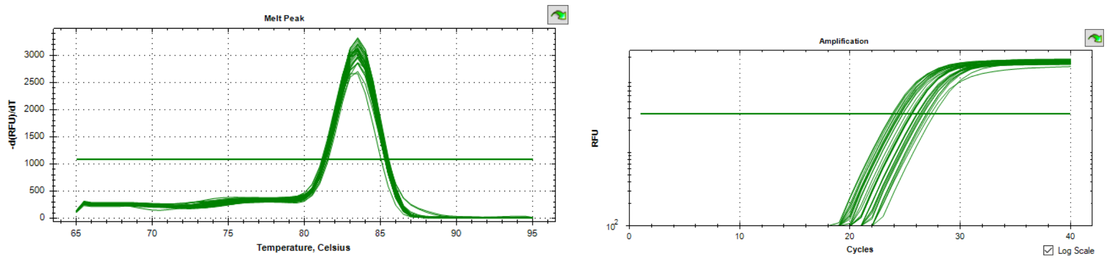

CYCD31

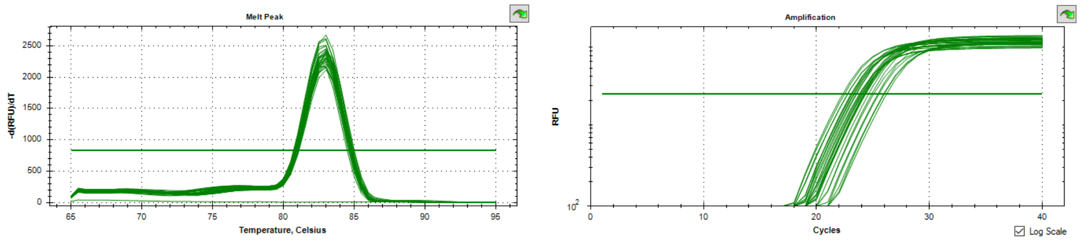

BG3

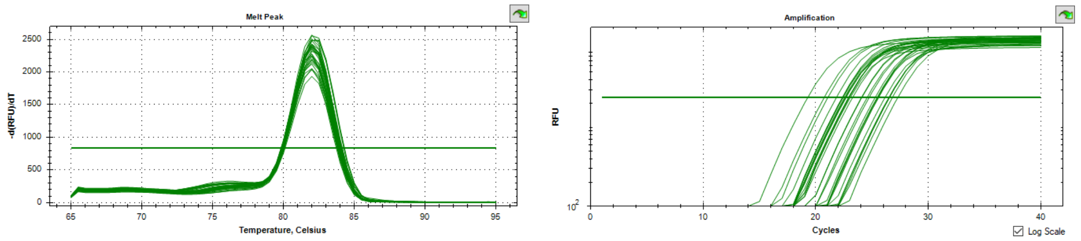

CAT2

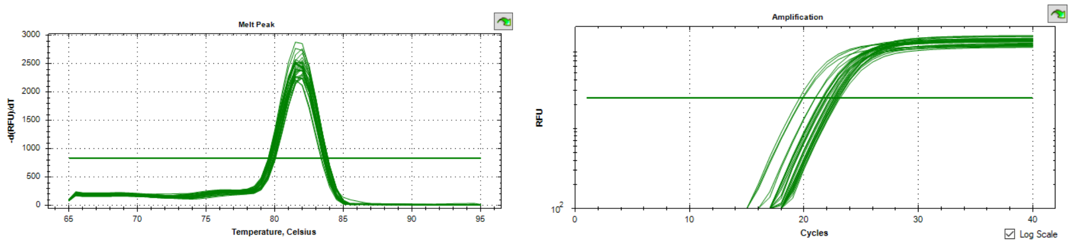

EXPA6

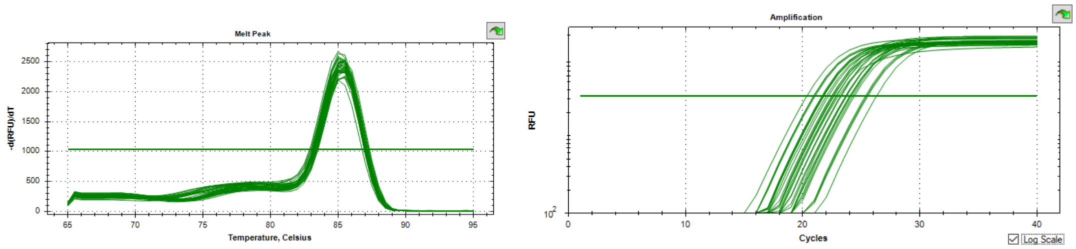

EXPLA2

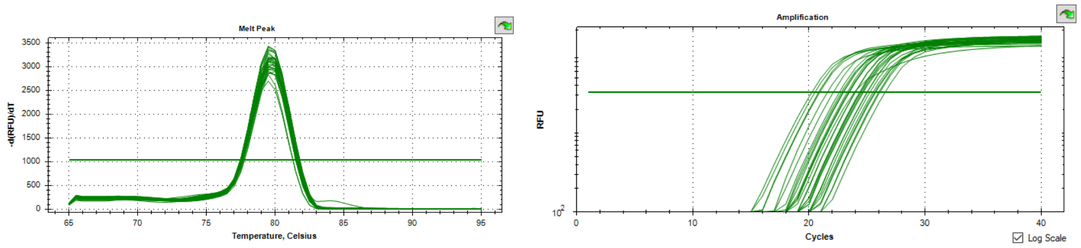

CDKB

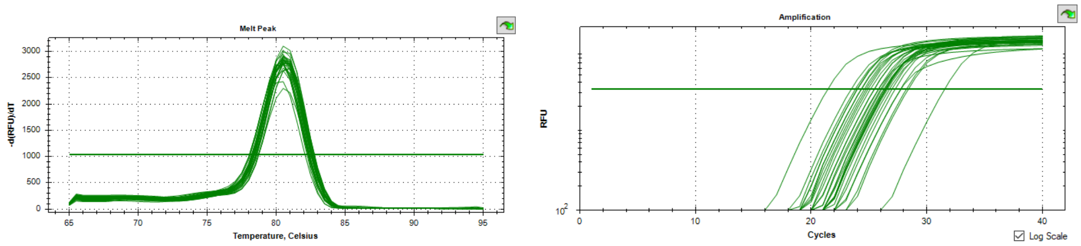

TUBA

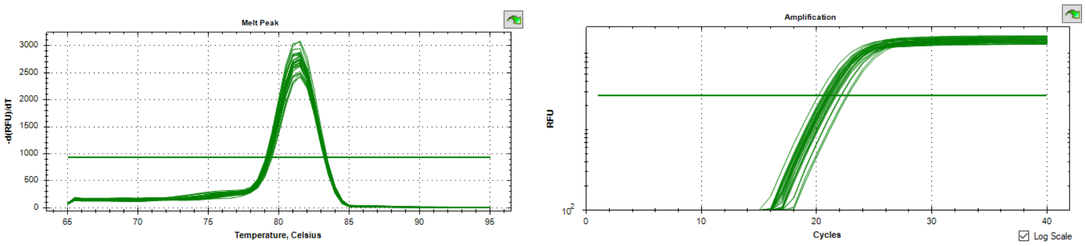

GRXS17

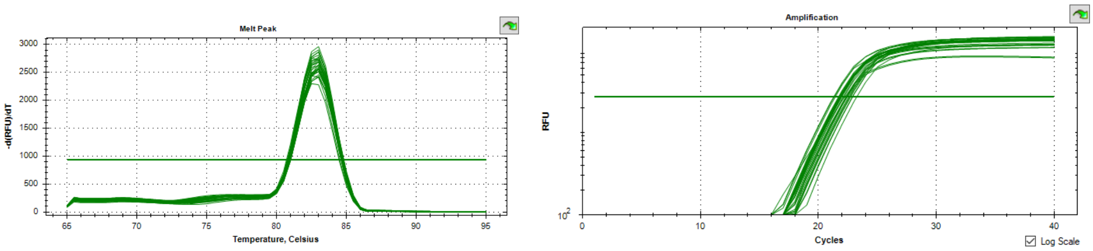

HIS1

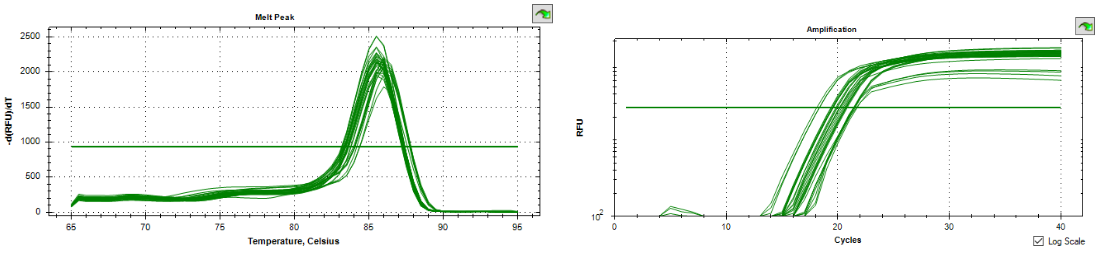

PER52

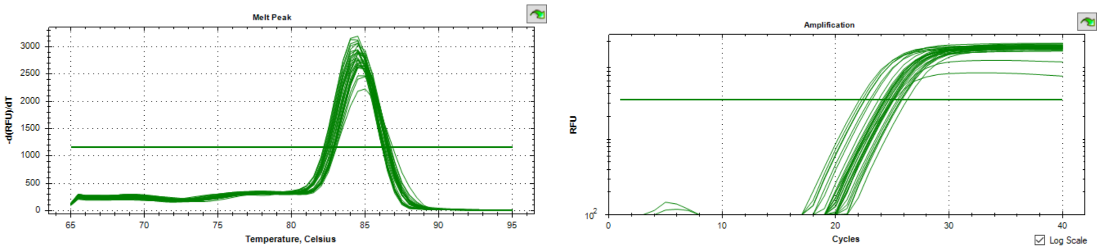

ARP6

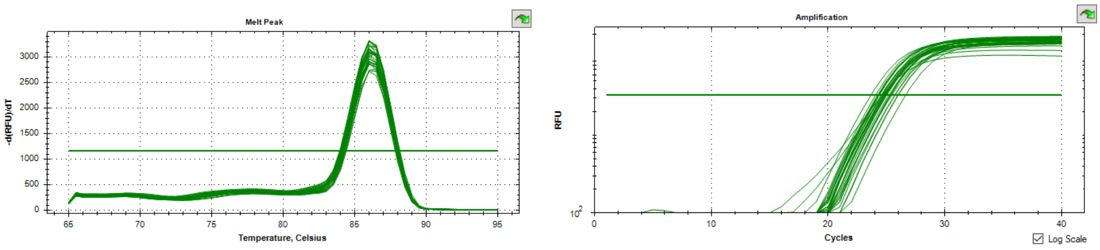

HSP70

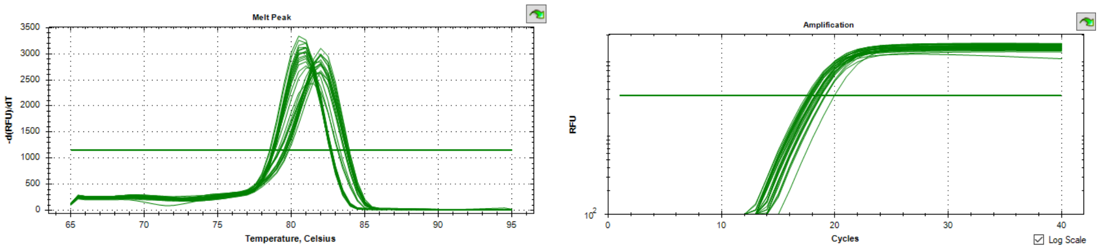

OSM34

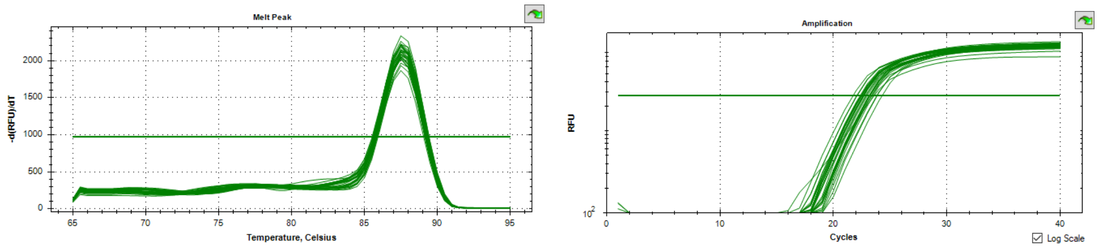

LOS1

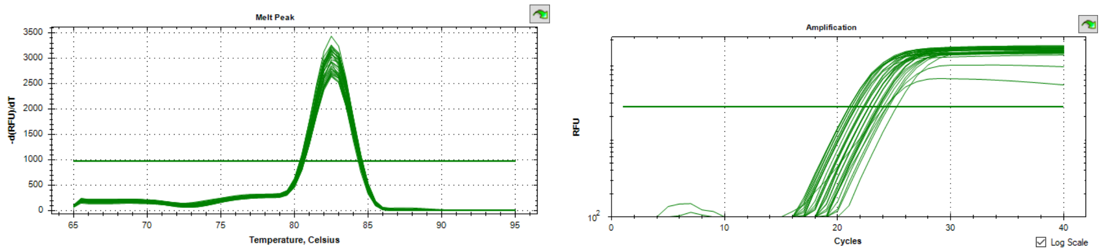

COR413

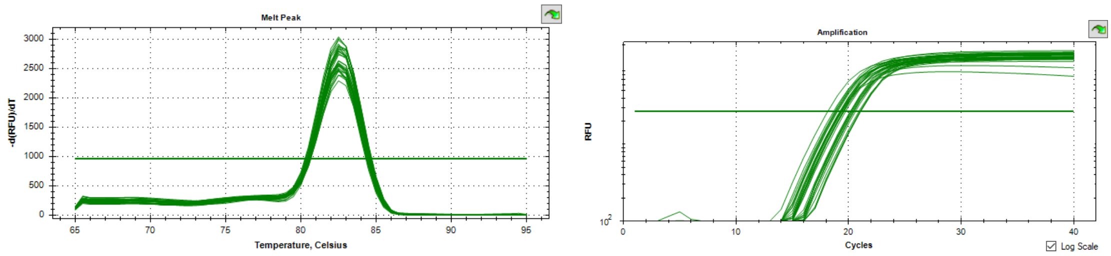

WORKY

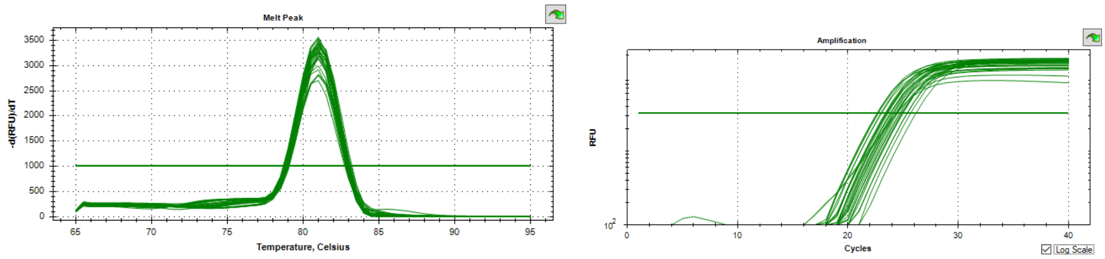

LEA

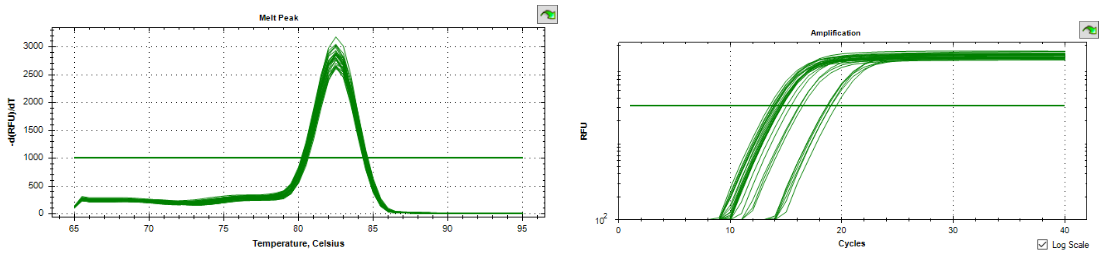

PHYA

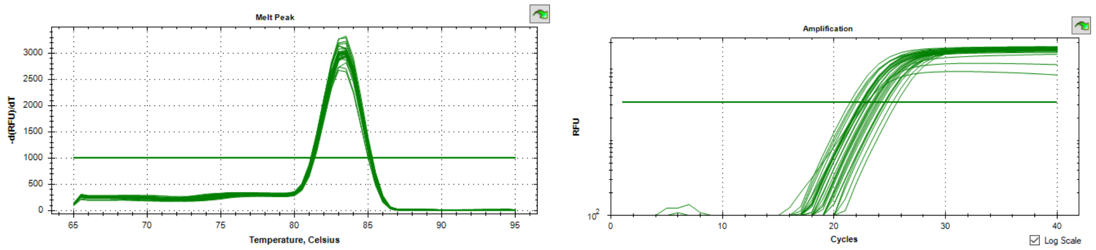

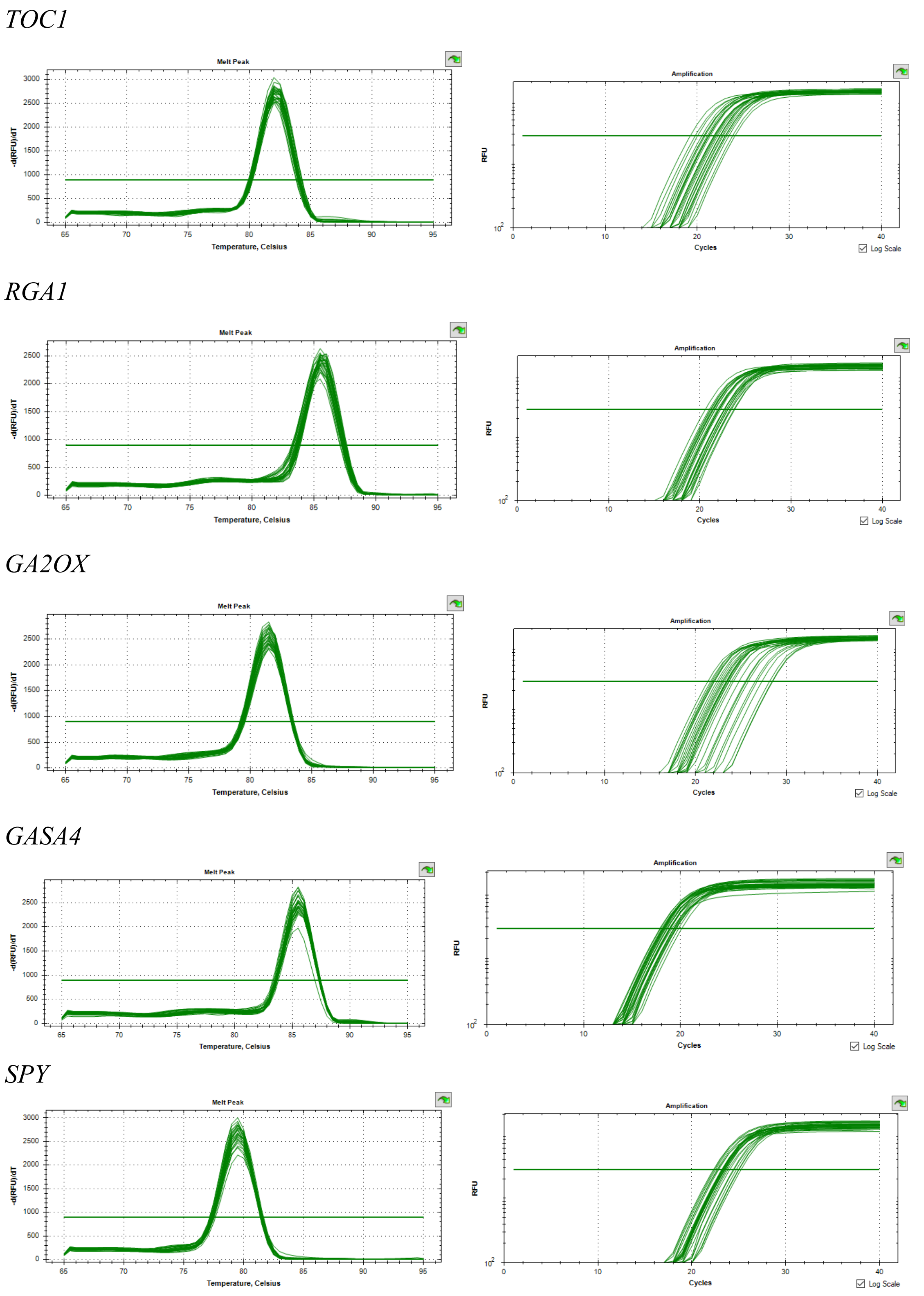

Figure. 5 | PCR amplification curves and melt curves of each pair of primer in this study. Both amplification and melt curves of each pair of primer contain three biological replicates of the two cultivars in the five move dates during bud endodormancy release. RFU, Relative Fluorescence Unit.
