# Supplementary material for: Comparative Study on Physiological Responses and Gene Expression of Bud Endodormancy Release Between Two Herbaceous Peony Cultivars (Paeonia lactiflora Pall.) With Contrasting Chilling Requirements
Source: Front Plant Sci. 2022 Feb 2;12:772285. doi: 10.3389/fpls.2021.772285 (PMC8847303; doi:10.3389/fpls.2021.772285)
Supplement: Supplementary file 5 [file Data_Sheet_2.DOCX]

**Supplementary Methods S1.**

**Carbohydrate Concentration Measurements**

The samples used for measuring the starch and soluble sugar contents were ground into fine powder, 300 mg subsamples were transferred to 15 mL centrifuge tubes and 5 mL of 80% ethanol (v/v) was added. Then, the homogenate was transferred to a 10 mL centrifuge tube and centrifuged at 4°C and 4000 rpm centrifugation for 5 min. The supernatant was collected and the extraction was repeated three times. The sugar extract was then diluted to 50 ml with distilled water and the soluble sugars contents were measured as described by Zhang et al. (2021a). The residues left in the centrifuge tubes after extracting sugars were dried at 80 °C for starch extraction using perchloric acid following the method described by Zhang et al. (2021a).

**Supplementary Methods S2.**

**Endogenous Hormone Quantitations**

Endogenous hormone levels of buds were determined by enzyme-linked immunosorbent assay (ELISA). Samples of buds were ground in a mortar at 0 °C in 10 ml of 80% (v/v) methanol extraction medium containing 1 mM butylated hydroxy-toluene as an antioxidant. The extract was incubated at 4 °C for 4 h and centrifuged at 4800 g for 15 min at 4 °C. The supernatants were sequen-tially passed through Chromosep C18 columns (C18 Sep-Park Cartridge, Waters Corp, Milford, MA, USA), pre-washed with 10 mL of 100% and 5 mL of 80% methanol. For detailed methods, please refer to Yang et al. (2001).
