# Supplementary figures and images for: Comparative Study on Physiological Responses and Gene Expression of Bud Endodormancy Release Between Two Herbaceous Peony Cultivars (Paeonia lactiflora Pall.) With Contrasting Chilling Requirements
Source: Front Plant Sci. 2022 Feb 2;12:772285. doi: 10.3389/fpls.2021.772285 (PMC8847303; doi:10.3389/fpls.2021.772285)

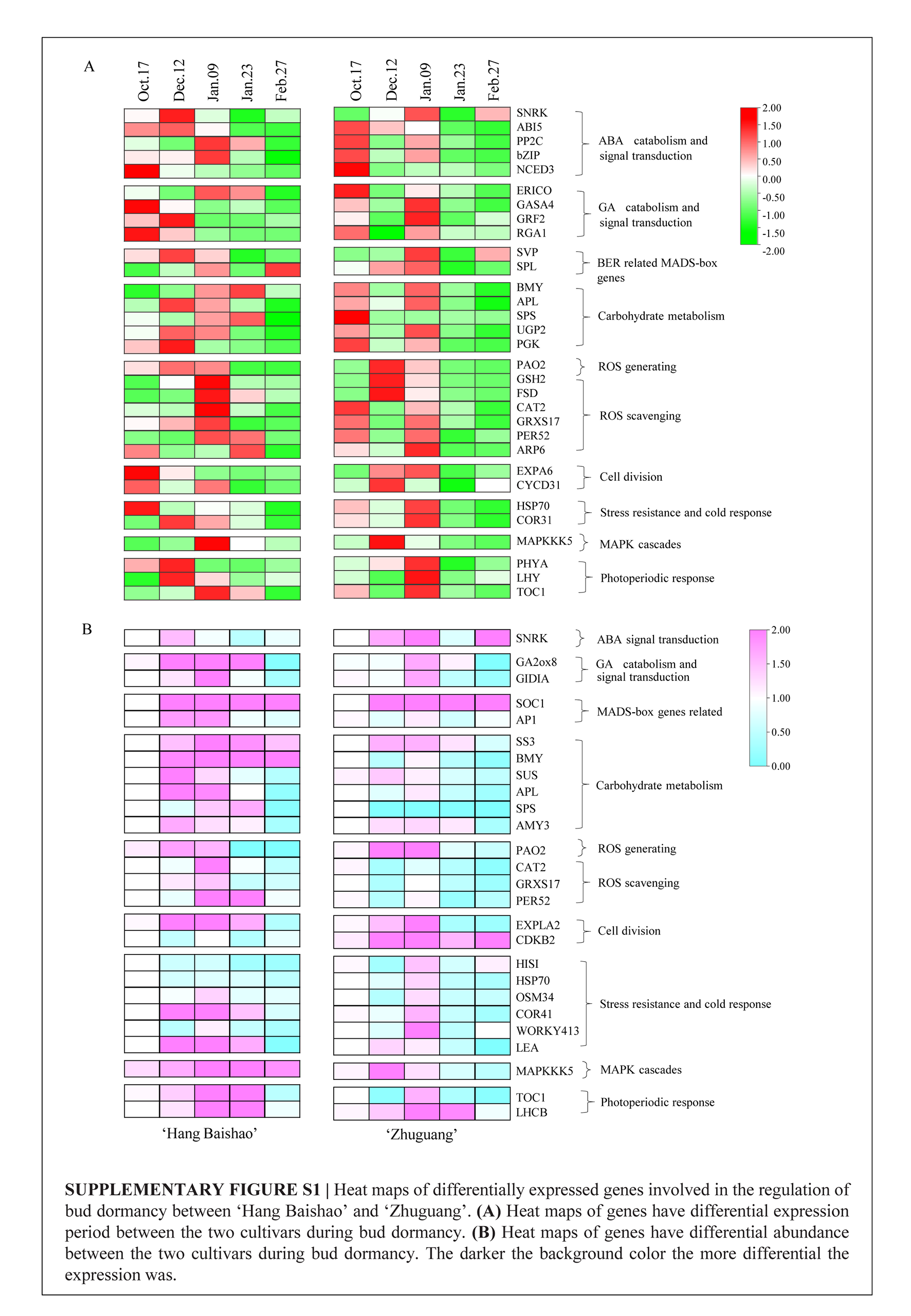

Supplement: Supplementary file 6 [file Image_1.TIF]

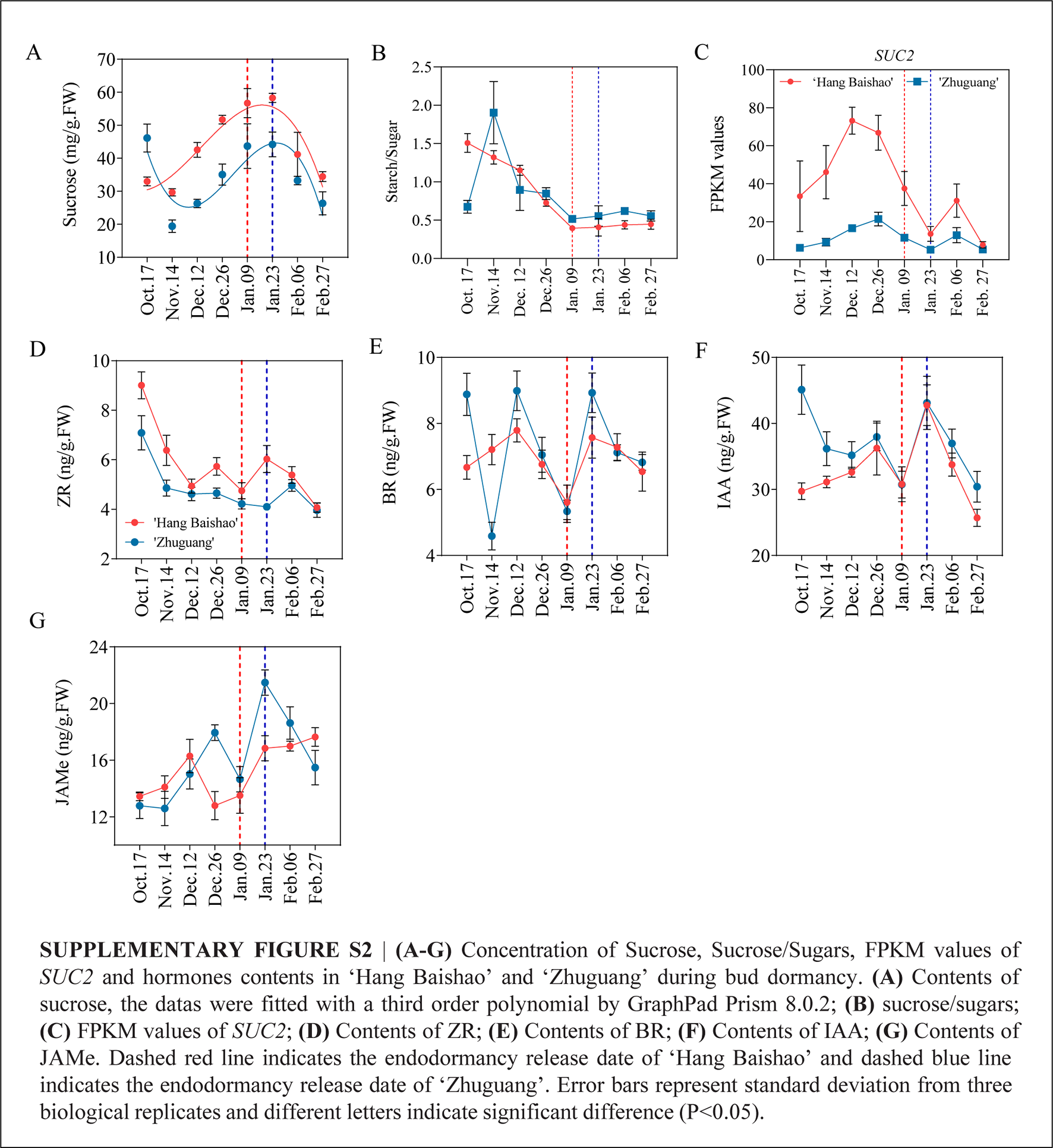

Supplement: Supplementary file 7 [file Image_2.TIF]
